# Schizophrenia is associated with altered DNA methylation variance

**DOI:** 10.1038/s41380-024-02749-5

**Published:** 2024-09-13

**Authors:** Dylan J. Kiltschewskij, William R. Reay, Murray J. Cairns

**Affiliations:** 1 School of Biomedical Sciences and Pharmacy, The University of Newcastle, Callaghan, NSW Australia; 2 Precision Medicine Program, Hunter Medical Research Institute, New Lambton, NSW Australia; 3 Menzies Institute for Medical Research, Hobart, TAS Australia

**Keywords:** Genetics, Molecular biology, Psychology

## Abstract

Varying combinations of genetic and environmental risk factors are thought to underpin phenotypic heterogeneity between individuals in psychiatric conditions such as schizophrenia. While epigenome-wide association studies in schizophrenia have identified extensive alteration of mean DNA methylation levels, less is known about the location and impact of DNA methylation variance, which could contribute to phenotypic and treatment response heterogeneity. To explore this question, we conducted the largest meta-analysis of blood DNA methylation variance in schizophrenia to date, leveraging three cohorts comprising 1036 individuals with schizophrenia and 954 non-psychiatric controls. Surprisingly, only a small proportion (0.1%) of the 213 variably methylated positions (VMPs) associated with schizophrenia (Benjamini-Hochberg FDR < 0.05) were shared with differentially methylated positions (DMPs; sites with mean changes between cases and controls). These blood-derived VMPs were found to be overrepresented in genes previously associated with schizophrenia and amongst brain-enriched genes, with evidence of concordant changes at VMPs in the cerebellum, hippocampus, prefrontal cortex, or striatum. Epigenetic covariance was also observed with respect to clinically significant metrics including age of onset, cognitive deficits, and symptom severity. We also uncovered a significant VMP in individuals with first-episode psychosis (*n* = 644) from additional cohorts and a non-psychiatric comparison group (*n* = 633). Collectively, these findings suggest schizophrenia is associated with significant changes in DNA methylation variance, which may contribute to individual-to-individual heterogeneity.

## Introduction

Psychotic disorders are associated with vast heterogeneity in terms of onset, progression, severity, and treatment response [1–3]. This phenotypic variability remains a major challenge for improving patient outcomes, necessitating precision medicine strategies which leverage an individual’s unique combination of molecular and environmental risk factors. Genetic risk factors are proving particularly crucial, given that schizophrenia is highly heritable and exhibits broad polygenic architecture, encompassing unique combinations of common variants, with individually small effect sizes but collectively large impact, in addition to rare and structural variants, some of which are highly deleterious [4, 5]. Mounting evidence suggests schizophrenia and psychosis also possess a strong epigenetic component, characterised by dysregulation of DNA methylation at CpG dinucleotides across the genome, which in some cases co-localise with loci prioritised by genome-wide association studies (GWAS) [6–12]. Indeed, epigenome-wide association studies (EWAS) have uncovered altered DNA methylation at genes previously implicated in these conditions, such as *SLC6A12*, *GABB1R* and *CACNA1C*, and in association with measures of progression and severity, including treatment resistance and cognitive deficits [6–12]. Since DNA methylation can substantially impact transcription and post-transcriptional regulation [13, 14], its dysregulation in psychiatric illness may therefore contribute to altered brain structure, connectivity, and cognition pertinent to these conditions. However, it is unclear whether epigenetic dysregulation also contributes to heterogeneity in schizophrenia and psychosis, necessitating further exploration with a view of identifying loci which inform molecular subtypes and precision medicine.

While EWAS are highly useful for ascertaining differentially methylated positions (DMPs) that may underpin mechanisms related to schizophrenia and psychosis, these studies are restricted to broad changes at the population level and do not address the immense molecular heterogeneity which may account for individual-to-individual phenotypic variation [15]. This is particularly important since DNA methylation is influenced by genetic variants and dynamically changes in response to environmental exposures, therefore, potentially serving as an important interface between genetic and environmental risk [16, 17]. Recent epigenetic scoring approaches, analogous to polygenic scoring using genetic variants, have successfully utilised EWAS summary statistics to quantify an individual’s total epigenetic risk in a manner that is associated with diagnostic status and treatment resistance [6, 18, 19]. However, few studies have examined epigenome-wide patterns of DNA methylation variance to identify variably methylated positions (VMPs) that potentially capture an individual’s lifetime exposure to environmental risk factors and contribute to molecular heterogeneity in a manner that can be reconciled with specific genes [15]. To address this, we conducted the largest epigenome-wide meta-analysis of DNA methylation variance in schizophrenia and first episode psychosis (FEP) to date, utilising four of the largest publicly available case-control cohorts [7]. Our analyses revealed vast patterns of variable DNA methylation in association with schizophrenia, which exhibit enrichment amongst genes pertinent to neuronal function, correlate with features of this disorder, and affect known schizophrenia-associated genes. Furthermore, we report divergence between DNA methylation variance and mean effects, which may contribute to heterogeneity in this disorder.

## Methods

### Cohort descriptions

Blood DNA methylation data was obtained from four case-control cohorts previously analysed in [7]: the University College London cohort (UCL [20]; *n*_*Controls*_ = 433, *n*_*Schizophrenia*_ = 414), the Irish Schizophrenia Genomics Consortium Dublin cohort (DUB [21]; *n*_*Controls*_ = 349, *n*_*Schizophrenia*_ = 364), the King’s College London Institute of Psychiatry, Psychology and Neuroscience cohort (IoPPN [22, 23]; *n*_*Controls*_ = 203, *n*_*Schizophrenia*_ = 290, *n*_*FEP*_ = 307) and the European Network of National Schizophrenia Networks Studying Gene-Environment Interactions cohort (EU-GEI [24], *n*_*Controls*_ = 521_,_ *n*_*FEP*_ = 413). For the UCL cohort, schizophrenia diagnoses were assigned via ICD-10 criteria and confirmed with the Lifetime Version of the Schedule for Affective Disorders and Schizophrenia, whereas nonpsychiatric controls were interviewed to exclude individuals with personal history of a Research Diagnostic Criteria-defined mental disorder or a family history of schizophrenia, bipolar disorder, or alcohol dependence [25]. Schizophrenia cases in the DUB cohort were assessed using DSM-IV criteria for schizophrenia or related disorders (schizoaffective or schizophreniform disorder), while controls were obtained from the Irish GeneBank. For the IoPPN cohort, participants with schizophrenia (sourced from the Improving Physical Health and Reducing Substance Use in Severe Mental Illness (IMPACT) study) were diagnosed via ICD-10 criteria, FEP (sourced from in-patient units of the South London and Maudsley Mental Health NHS Foundation Trust) was diagnosed as per ICD-10 criteria with validation via Schedules for Clinical Assessment in Neuropsychiatry, and controls were required to pass the Psychosis Screening Questionnaire [26]. Finally, FEP cases in the EU-GEI cohort were diagnosed via ICD-10 criteria, excluding individuals meeting criteria for organic psychosis (ICD-10: F09) or diagnosed with transient psychotic symptoms from acute intoxication (ICD-10: F1X.5). Controls required absence of a diagnosis, and/or treatment for, a psychotic disorder.

After quality control (see below), final sample sizes were as follows: UCL: *n*_*Controls*_ = 432, *n*_*Schizophrenia*_ = 414; DUB: *n*_*Controls*_ = 322, *n*_*Schizophrenia*_ = 339; IoPPN: *n*_*Controls*_ = 200, *n*_*Schizophrenia*_ = 283, *n*_*FEP*_ = 294; EU-GEI: *n*_*Controls*_ = 433, *n*_*FEP*_ = 350. In the IoPPN cohort, higher Horvath epigenetic age was observed amongst individuals with schizophrenia compared to controls (mean difference = 11.61 years, *P* = 2.74 × 10^–36^, *t*-test), while individuals with FEP in the EU-GEI cohort exhibited lower Horvath age (mean difference = 4.72 years, *P* = 2.83 × 10^–16^, *t*-test; Table 1). This metric was used instead of reported age due to varying degrees of missingness of reported age, noting there is no evidence for age acceleration within these cohorts [7]. There was weak evidence suggesting males were overrepresented in the IoPPN schizophrenia (*χ*^*2*^ = 4.51, *P* = 0.03, *df* = 1, *χ*^*2*^ test) and FEP (*χ*^*2*^ = 3.84, *P* = 0.05, *df* = 1, *χ*^*2*^ test) groups relative to controls, while there was comparatively stronger evidence of male overrepresentation amongst individuals with FEP in the EU-GEI cohort (*χ*^*2*^ = 24.5, *P* = 7.29 × 10^–7^, *df* = 1, *χ*^*2*^ test, Table 1). All participants were predominantly of white European ancestry, noting that some ancestral heterogeneity was present within the IoPPN and EU-GEI cohorts. However, covarying for up to five genetic principal components (PCs) has previously demonstrated little impact on EWAS results for these cohorts [7].

**Table 1:** Cohort demographics.

		UCL (GSE84727)	DUB (GSE147221)	IoPPN (GSE152027)	IoPPN (GSE152027)	EU-GEI (GSE152026)
**Phenotype**		Schizophrenia	Schizophrenia	Schizophrenia	FEP	FEP
***N*** **(post QC)**	*n*_*Controls*_	432	322	200	200	433
	*n*_*Cases*_	414	339	283	294	350
**Predicted sex**	*n*_*Controls, male*_	319	233	113	113	203
	*n*_*Controls, female*_	113	89	87	87	230
	*n*_*Cases, male*_	283	249	188	193	227
	*n*_*Cases, female*_	131	90	95	101	123
***χ***^***2***^ **test (case vs control)**	*χ*^*2*^*-*statistic	2.838	0.052	4.508	3.845	24.538
	*P*-value	0.092	0.820	0.034	0.050	7.29 x 10^–7^
**Mean (*SD*) Horvath age (years)**	Controls	54.33 (9.90)	50.06 (9.56)	44.01 (9.09)	44.01 (9.09)	46.61 (8.33)
	Cases	54.21 (11.2)	50.86 (9.82)	55.63 (9.07)	43.45 (8.97)	41.89 (7.45)
**Mean difference**		–0.13	0.80	11.61	–0.56	–4.72
***t*-test (case vs control)**	*t*-statistic	0.176	–1.061	–13.846	0.674	8.363
	*P*-value	0.860	0.288	2.74 x 10^–36^	0.501	2.83 x 10^–16^

### Quantification of DNA methylation

Genomic DNA sampled from blood was previously subjected to bisulfite conversion via EZ-96 DNA methylation kit (Zymo Research, CA, USA) and quantification via Illumina Infinium HumanMethylation450 BeadChip (Illumina Inc, CA, USA; UCL, DUB and IoPPN) or Infinium HumanMethylationEPIC BeadChip (EU-GEI) [7]. Raw signal intensities and detection *P*-values were obtained from the Gene Expression Omnibus (UCL: GSE84727, DUB: GSE147221, IoPPN: GSE152027, and EU-GEI: GSE152026) and subjected to processing and quality control measures similar to those employed in [7]. Methylated and unmethylated intensities were converted into a “MethylSet” object using the *minfi R* package (v1.44.0) [27], after which we identified and omitted: (1) fully methylated control samples, (2) samples flagged as poor quality by the original authors, (3) samples in which >10% of probes exhibited a detection *P*-value > 0.01, (4) probes with a detection *P* > 0.01 in >10% of samples, (5) samples with a median methylated and/or unmethylated signal intensity <2500 units, and (6) outlier samples identified via the *outlyx* command (default parameters) from the *wateRmelon* package (v.1.35.2) [28] (Fig. S1). Sex was then estimated, and individuals with mismatched reported and estimated sex, or undefined predicted sex, were excluded (Fig. S1). Individuals with no reported sex, but conclusive estimated sex were retained. Non-autosomal probes were subsequently excluded, as well as non CpG probes, cross hybridising probes (as per [29–31]), and probes within 5nt of a single nucleotide polymorphism with minor allele frequency >1% in Europeans (as per [32]). Samples were then normalised via Dasen method [28], and beta distributions were visually assessed (Fig. S2) Finally, methylation-derived age (Horvath) and cell type proportions were estimated via *wateRmelon*, and methylation-predicted smoking scores were derived using *EpiSmokeR* (v0.1.0) [33].

### Within-cohort epigenome-wide association studies

Before examining DNA methylation variance, probe beta values were residualised on sentrix identifiers, sentrix positions and methylation-derived age, sex, smoking scores and cell type proportions. VMPs associated with diagnostic status were subsequently examined via Levene’s Test, which assesses equality of variances amongst categorical variables in a manner robust to departures from normality [34]. Two supplementary tests were employed for comparison, including Bartlett’s Test, which is more sensitive than Levene’s Test but assumes normality [34], and the Fligner-Killeen Test, which is highly robust to departures from normality at the cost of sensitivity [35]. For all three tests, we employed the *bacon R* package (v.1.20.0) [36] to control for *P*-value inflation utilising a Bayesian method, with signed *Z*-scores used as input.

EWAS examining mean effects were additionally conducted in each cohort via multiple linear regression of diagnostic status against probe beta values, covaried for biological and technical confounders listed above. All results were adjusted using the *bacon* package, utilising effect sizes (*β*) and their standard errors (*SE*) as input. To assess the consistency of our results versus those reported by Hannon et al. [7], we examined univariate correlation of *Z*-scores (*β* / *SE*) between both studies using probes previously associated with schizophrenia (1 048 probes) or FEP (95 probes), revealing strong correlation across all cohorts (Pearson *r* ≥ 0.81, *P* ≤ 2.2 × 10^–16^, Fig. S3).

### Meta-analysis

Variance effects were meta-analysed via Stouffer’s method utilising signed *Z*-scores derived from Levene’s Test, weighted by the square-root of the sample size for each study (similar to [37]). This methodology was employed since Levene’s Test only produces *P*-values, rather than effect sizes and standard errors. For each phenotype, probes reported in at least two studies were examined, yielding 416,956 probes for the schizophrenia meta-analysis and 310,019 for FEP. Probes surpassing a Benjamini-Hochberg False Discovery Rate (*FDR*_*BH*_) < 0.05 were defined as VMPs, whereas probes surpassing a more conservative, Bonferroni-corrected *P* < 1.2 × 10^–7^ (schizophrenia, 416,956 tests) or *P* < 1.61 × 10^–7^ (FEP, 310,019 tests) were deemed epigenome-wide significant.

Mean effects were additionally subjected to fixed and random effects meta-analyses utilising the inverse variance weighted method (IVW), implemented via the *metagen* function of the *meta R* library (v6.5.0) [38]. Random effects meta-analyses were used as the principal model for both phenotypes. Adjusted probe effect sizes and their standard errors were used as input (as per [7]), with probes surpassing an *FDR*_*BH*_ < 0.05 deemed DMPs, while probes surpassing the Bonferroni thresholds (same as the VMP meta-analysis) were defined as epigenome-wide significant.

### Analysis of DNA methylation in post-mortem brain

Five publicly available datasets were used to analyse DNA methylation profiles within the post-mortem cerebellum (GSE89702 [9], *n*_*Controls*_ = 17, *n*_*Schizophrenia*_ = 16), hippocampus (GSE89703 [9], *n*_*Controls*_ = 13, *n*_*Schizophrenia*_ =14), prefrontal cortex (GSE74193 [39], *n*_*Controls*_ = 97, *n*_*Schizophrenia*_ = 104) and striatum (GSE89705 [9], *n*_*Controls*_ = 17, *n*_*Schizophrenia*_ = 16; GSE89706 [9], *n*_*Controls*_ = 28, *n*_*Schizophrenia*_ = 21). All studies were processed as detailed above, noting only autosomal probes were available for data obtained from [9], thus sex could not be estimated (See Fig. S4 for outliers and beta distributions). For each data set, VMPs and DMPs were identified in association with schizophrenia after controlling for sentrix identifiers, sentrix positions, reported age and sex, and methylation-derived smoking status and cell-type proportions (NeuN positive and negative). Reported age was utilised since this information was available for all participants. Post-mortem intervals were not reported and therefore could not be included as covariates. Methylation profiles from the striatum were meta-analysed as outlined above to enhance sample size.

### Phenome-wide epigenetic covariance

Phenome-wide epigenetic covariance was explored between schizophrenia associated DNA methylation profiles and traits/exposures from *EWASCatalog* [40], a repository of > 7000 EWAS, reporting probes with *P*_*EWAS*_ < 1 × 10^–4^. Pearson correlation was examined between meta-analysis *Z*-scores and *EWASCatalog* effect sizes, the latter of which were scaled to have zero mean and unit variance (i.e. converted to *Z*-scores). Our analyses were restricted to traits with ≥ 10 reported probes, European ancestry, and covariates for age, sex, technical confounders, smoking, and cell-type proportions, noting that only mean effects are reported in this repository.

## Results

### Variably methylated loci associated with schizophrenia and first episode psychosis

**Fig. 1:**
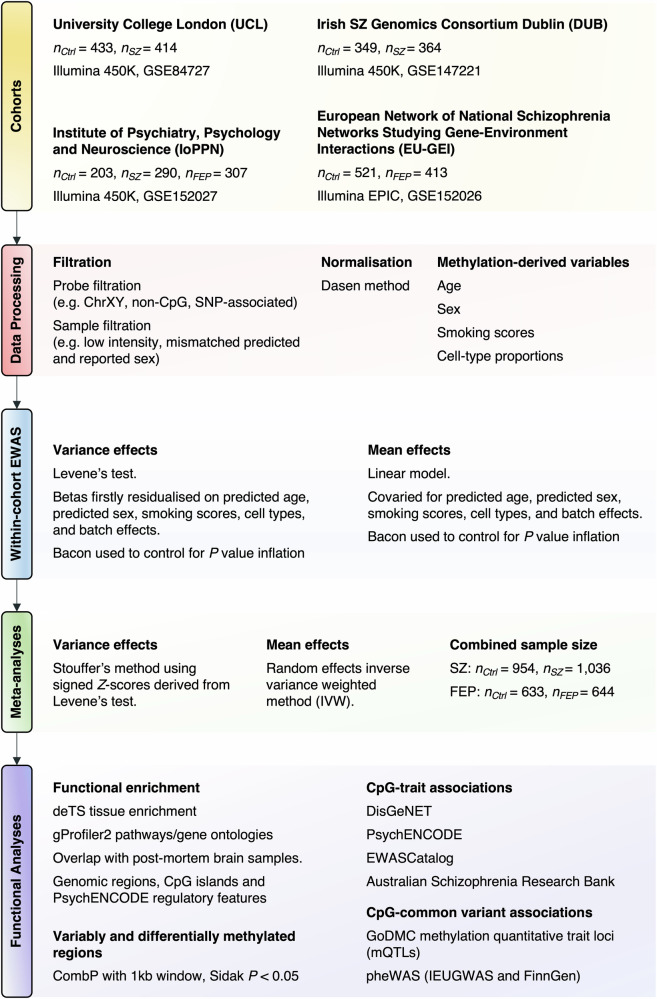
Exploring patterns of DNA methylation variance in schizophrenia and first-episode psychosis. DNA methylation quantified from the blood of individuals with schizophrenia, first episode psychosis or non-psychiatric controls from four independent cohorts was obtained from the gene expression omnibus. After initial data processing, quality control, normalisation and calculation of methylation-derived variables, epigenome-wide association studies examining DNA methylation variance or mean effects between cases and controls were conducted within each cohort and subsequently meta-analysed. Significant probes were then subjected to a battery of functional analyses examining: enrichment across tissues, pathways, gene ontologies and genomic regions, overlap with brain DNA methylation patterns, variably and differentially methylated regions, enrichment amongst genes previously associated with psychiatric illness, epigenetic covariance with other traits and phenotypes, and interplay with common genetic variants.

**Fig. 2:**
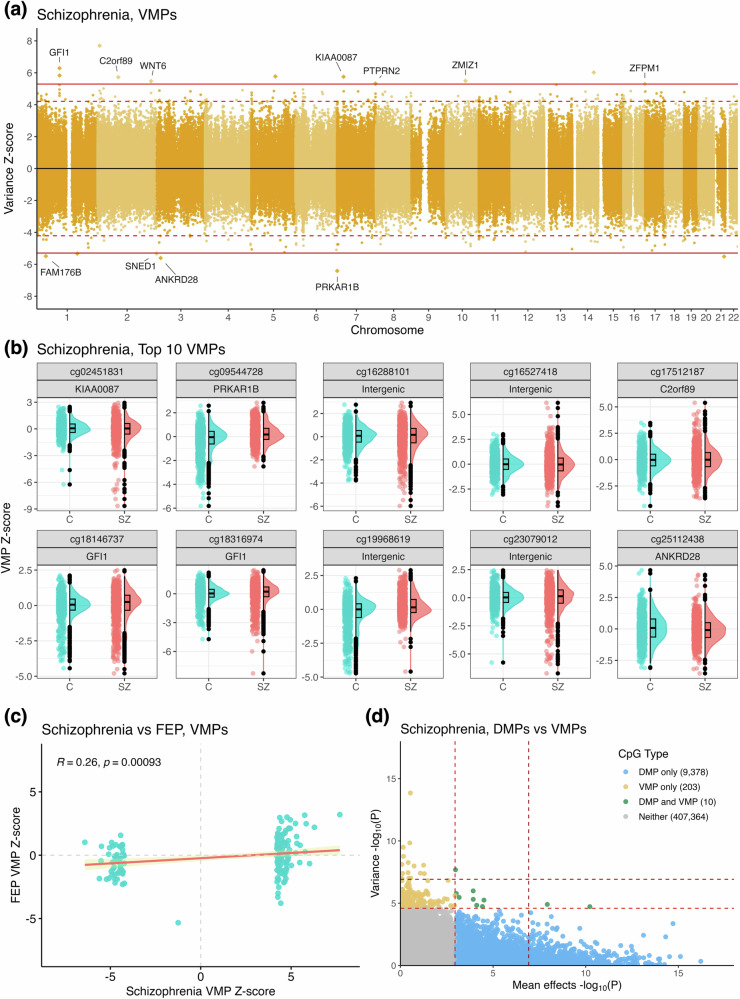
Variably methylated positions associated with schizophrenia. **a** Miami plot depicting the epigenome-wide distribution of meta-analysis *Z*-scores for DNA methylation variance associated with schizophrenia. Positive *Z*-scores denote sites with increased variance in schizophrenia, while negative *Z*-scores denote sites with decreased variance. Solid red line indicates the threshold for epigenome-wide significance (*P* < 1.2 x 10^–7^), whereas the dashed red line represents the Benjamini-Hochberg FDR. **b** Raincloud plots presenting the top 10 VMPs associated with schizophrenia, ranked by meta-analysis *P*-value. C = control, SZ = schizophrenia. All sites are labelled with their nearest gene as per the IlluminaHumanMethylation450K manifest. **c** Univariate correlation of meta-analysis *Z*-scores for VMPs associated with schizophrenia (213 VMPs) and FEP (1 VMP). Pearson correlation coefficient and associated *P*-value reported top left. **d** Comparison of –log_10_ meta-analysis *P*-values for schizophrenia associated mean effects (x-axis) and variance (y-axis), revealing minimal overlap between VMPs and DMPs.

To explore epigenetic heterogeneity in schizophrenia and FEP, we conducted the largest epigenome-wide meta-analysis of DNA methylation variance in association with these psychiatric conditions (See Fig. 1 for a methodological overview). A total of 213 VMPs were identified in association with schizophrenia, of which 17 surpassed the Bonferroni threshold for epigenome-wide significance (Fig. 2a, b, Table 2, Table S1). Overall, a modest bias towards elevated variance was uncovered, wherein 139 VMPs (65.3%) exhibited increased variance in schizophrenia. In contrast, a single epigenome-wide significant VMP (cg17339327, *P* = 9.91x10^–8^, *Z* = –5.33) was associated with FEP, residing within the schizophrenia associated *STK19* and *DXO/DOM3Z* genes (Fig. S6, Table S2). Aggregation of VMPs into spatially localised variably methylated regions (VMRs) revealed 57 significant (*P*_*Sidak*_ < 0.05, *n*_*probes*_ ≥ 2) VMRs for schizophrenia and 10 VMRs for FEP (Fig. S6, Tables S3, S4; see Supplementary Methods). In both cases, VMR functional enrichment (as described herein for VMPs) was sparse, however some schizophrenia associated VMRs overlapped genes pertinent to neuronal function, such as *ADBR3*, *GLRA1*, *GRM2*, *HTR2A*, *KCNK10*, *KCNQ1* and *S100P*, while the strongest association involved 6 VMPs overlapping *GFI1*, a gene associated with haematopoiesis and apoptosis [41, 42] (Fig. S6, Tables S3–S9). For FEP, particularly strong association was identified for a VMR within *RNF39* (15 VMPs), encoding a ring finger protein that is expressed in glutamatergic neurons and associated with long term potentiation [43] (Fig. S6, Table S4).

**Table 2:** Epigenome-wide significant VMPs associated with schizophrenia.

				Variance meta-analysis (Stouffer’s method)							Mean effects meta-analysis (IVW_RE_)			
Probe	Locus	Nearest gene	Genic location	^1^*Z*_*Levene*_	^1^*P*_*Levene*_	^2^*Z*_*Bartlett*_	^2^*P*_*Bartlett*_	^3^*Z*_*FK*_	^3^*P*_*FK*_	^4^Direction	^5^*β*_*IVW-RE*_	^5^*SE*_*IVW-RE*_	^5^*P*_*IVW-RE*_	^6^Direction
cg23079012	chr2:8343710			7.69	1.42 x 10^–14^	10.41	2.15 x 10^–25^	7.63	2.31 x 10^–14^	+++	−0.21	0.20	0.29	−−−
cg09544728	chr7:601503	*PRKAR1B*	Gene body	−6.41	1.44 x 10^−^^10^	−5.21	1.91×10^–7^	−6.19	5.87 x 10^–10^	+−−	1.77	1.75	0.31	−++
cg18316974	chr1:92947035	*GFI1*	Gene body	6.29	3.22 x 10^–10^	8.08	6.64 x 10^–16^	6.22	5.07 x 10^–10^	+++	0.14	0.39	0.71	++−
cg16288101	chr14:88621538			6.02	1.72 x 10^–9^	6.90	5.37 x 10^–12^	5.54	3.11 x 10^–8^	+++	−0.17	0.43	0.69	−+−
cg18146737	chr1:92946700	*GFI1*	Gene body	5.83	5.46 x 10^–9^	6.13	8.82 x 10^–10^	5.95	2.68 x 10^–9^	+++	0.48	0.55	0.38	++−
cg16527418	chr5:98175367			5.77	7.71 x 10^–9^	6.26	3.76 x 10^–10^	5.50	3.75 x 10^–8^	+++	0.10	0.48	0.83	++−
cg02451831	chr7:26578098	*KIAA0087*	Gene body	5.75	8.89 x 10^–9^	7.58	3.55 x 10^–14^	5.72	1.10 x 10^–8^	+++	−0.46	0.24	0.06	−−−
cg17512187	chr2:85082845	*C2orf89*	Gene body	5.73	1.03 × 10^–8^	5.49	3.97 x 10^–8^	5.65	1.56 x 10^–8^	+++	0.39	0.29	0.18	++−
cg25112438	chr3:15839498	*ANKRD28*	Gene body	−5.61	2.07 x 10^–8^	−4.16	3.15 x 10^–5^	−5.72	1.06 x 10^–8^	−−−	−0.25	0.08	0.00	−−−
cg19968619	chr21:42676391			−5.51	3.53 x 10^–8^	−5.28	1.31 x 10^–7^	−5.32	1.06 x 10^–7^	−−−	1.04	1.20	0.39	−+−
cg14371731	chr10:81003175	*ZMIZ1*	Gene body	5.50	3.88 x 10^–8^	4.80	1.62 × 10^–6^	5.89	3.93 x 10^–9^	+++	0.99	0.57	0.08	+++
cg13500072	chr1:36788387	*FAM176B*	Gene body	−5.49	4.07 x 10^–8^	−4.35	1.35 x 10^–5^	−5.47	4.40 x 10^–8^	−−−	0.08	0.25	0.76	+−+
cg00011225	chr2:219738314	*WNT6*	Gene body	5.48	4.25 x 10^–8^	5.42	5.87 x 10^–8^	5.69	1.25 x 10^–8^	+++	0.38	0.19	0.05	+++
cg02295023	chr1:165910942			−5.33	9.70 x 10^–8^	−3.89	1.02×10^–4^	−5.49	4.05 x 10^–8^	−−−	0.72	0.66	0.28	+++
cg11046579	chr2:241955055	*SNED1*	Gene body	−5.33	1.01 x 10^–7^	−4.99	5.94 x 10^–7^	−4.84	1.29 x 10^–6^	−−−	0.99	1.06	0.35	++−
cg11072645	chr7:157754746	*PTPRN2*	Gene body	5.32	1.02×10^–7^	4.05	5.10 x 10^–5^	5.34	9.29 x 10^–8^	+++	0.11	0.52	0.83	+−+
cg04983687	chr16:88558223	*ZFPM1*	Gene body	5.31	1.12×10^–7^	4.15	3.37×10^–5^	5.43	5.57×10^–8^	+++	0.19	1.42	0.89	+−+

^1^Stouffer’s *Z*-score and *P*-value from meta-analysis of within-cohort variance effects identified via Levene’s Test.

^2^Stouffer’s *Z*-score and *P*-value from meta-analysis of within-cohort variance effects identified via Bartlett’s Test.

^3^Stouffer’s *Z*-score and *P*-value from meta-analysis of within-cohort variance effects identified via Fligner-Killeen Test.

^4^Direction of variance in association with schizophrenia across the UCL, DUB and IoPPN cohorts, respectively.

^5^Effect size (*β*_*IVW-RE*_), standard error (*S*_*EIVW-RE*_) and *P*-value (*P*_*IVW-RE*_) from meta-analysis (random effects inverse variance method) of within-cohort mean effects.

^6^Direction of mean effects across the UCL, DUB and IoPPN cohorts, respectively.

Interestingly, comparison of VMP meta-analysis *Z*-scores between schizophrenia and FEP revealed positive correlation (Pearson *r* = 0.26, *P* = 9.3 x 10^–4^)_,_ suggesting there is some concordance between DNA methylation variance patterns amongst these conditions (Fig. 2c). To determine whether DNA methylation variance was also associated with mean effects, DMPs were identified for each condition as follows: schizophrenia: 9389 DMPs, 913 epigenome-wide significant; FEP: 271 DMPs, 10 epigenome-wide significant (Fig. S7, Tables S1, S2). Comparison of DMP and VMP meta-analysis *P-*values for schizophrenia revealed only 10 CpG sites (0.1% of all DMPs and VMPs) with both significantly altered mean and variance in the disorder, suggesting mean and variance effects were largely distinct (Fig. 2d). DMPs were also correlated between phenotypes, albeit to a lesser extent than VMPs (Pearson *r* = 0.18, *P* ≤ 2.2 × 10^–16^, Fig. S8).

### Schizophrenia VMPs are overrepresented amongst brain- and neuron-enriched genes

**Fig. 3:**
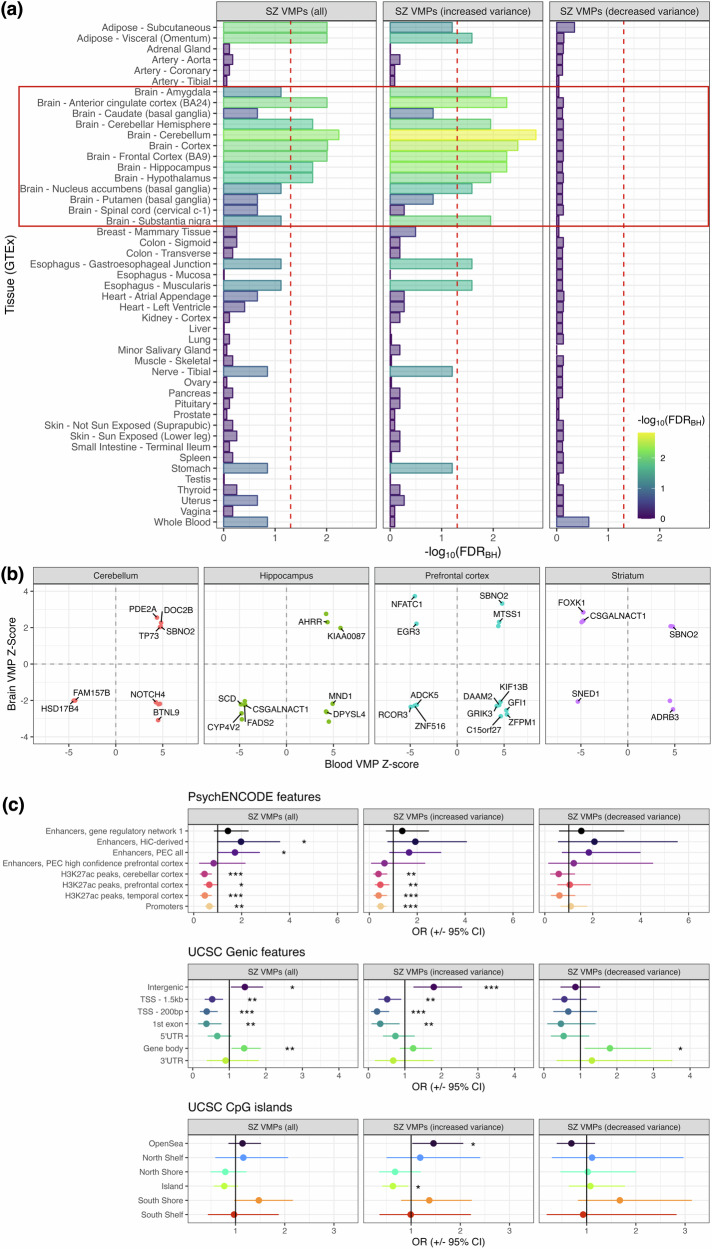
Tissue and genomic enrichment profiles for schizophrenia associated VMPs. **a** Overrepresentation of schizophrenia associated VMPs amongst tissue enriched gene expression profiles from GTEx (v7). For each tissue, genes expressed in the top 5% of all genes were deemed tissue enriched. Vertical red line denotes *FDR*_*BH*_ < 0.05, as determined via Fisher’s exact test. Red box denotes CNS tissues. **b** Scatter plots depicting schizophrenia associated VMPs from blood and their corresponding variance *Z*-scores in the cerebellum, hippocampus, prefrontal cortex, or striatum. All brain *Z*-scores surpassed a nominal *P* < 0.05. **c** Forest plots depicting enrichment of schizophrenia associated VMPs within genomic regulatory regions reported by *PsychENCODE* [54], gene-centric features from UCSC [98] and CpG islands and neighbouring regions from UCSC. Data presented as odds ratios ±95% confidence interval. * = *P* < 0.05, ** = *FDR*_*BH*_ < 0.05, *** = *P*_*Bonf*_ < 0.05.

We next examined whether schizophrenia associated VMPs identified from the blood disproportionately affected tissue-enriched genes from *GTEx* (v7) using the *deTS R* package (v1.0) [44]. Interestingly, these VMPs were overrepresented amongst genes enriched within several brain regions, including the anterior cingulate cortex, cerebellum, cortex, hippocampus and hypothalamus, as well as peripheral tissues, specifically subcutaneous and visceral adipose tissue (*FDR*_*BH*_ < 0.05, Fisher’s Exact Test; Fig. 3a, Table S10). These patterns were largely observed for VMPs with increased variance schizophrenia, whereas no significant results were uncovered for VMPs with decreased variance (Fig. 3a, Table S10). Using *gProfiler2* (v.0.2.2) [45], we also observed strong overrepresentation of neuronal cellular component gene ontology (GO) annotations such as *synapse*, *axon* and *neuronal cell body*, and molecular function terms relating to glutamatergic signalling, including *G protein-coupled glutamate receptor activity* and *group II metabotropic glutamate receptor activity*, which were again most strongly represented amongst VMPs with increased variance (Table S11). Similarly, schizophrenia associated DMPs exhibited enrichment amongst brain and peripheral tissues such as adipose, gastrointestinal and lung tissues, amongst others, while GO enrichment profiles were comparatively diverse (Fig. S9, Tables S10, S11).

### Detection of blood based VMPs within post-mortem brain

To explore whether schizophrenia associated VMPs sampled from the blood exhibited concordant changes in the brain, VMPs were profiled in the post-mortem cerebellum, hippocampus, prefrontal cortex, and striatum of affected individuals and non-psychiatric controls. While modest sample sizes restricted the identification of strong VMP signals after correction for multiple testing, 44 VMPs identified in blood exhibited nominal variance effects in the brain as follows: cerebellum: 10 VMPs, hippocampus: 13 VMPs, prefrontal cortex: 14 VMPs, striatum: 9 VMPs, noting that cg18608055 (*Z*_*Blood*_ = 4.79, *Z*_*Cerebellum*_ = 2.05, *Z*_*Prefrontal cortex*_ = 3.32, *Z*_*Striatum*_ = 2.05) was the only VMP represented in >1 region (Fig. 3b, Tables S12–S16). Interestingly, this CpG resides within *SBNO2*, a transcriptional co-regulator associated with cellular responses to interleukin-6 signalling in the CNS [46]. Blood and brain variance patterns were concordant for 23 VMPs, including a CpG residing within *DOC2B* (cg09464268, *Z*_*Blood*_ =4.83, *Z*_*Cerebellum*_ = 2.27), a calcium sensor associated with calcium-dependent neurotransmitter release in the absence of action potentials, and glucose-stimulated insulin secretion in the pancreas [47–50] (Fig. 3b, Table S16). A number of genes involved in fatty acid metabolism were also uncovered, including *HSD17B4* (cg17593721, *Z*_*Blood*_ = –4.49, *Z*_*Cerebellum*_ = –2.01), *CYP4V2* (cg24794857, *Z*_*Blood*_ = –4.79, *Z*_*Hippocampus*_ = –2.71), *FADS2* (cg00603274, *Z*_*Blood*_ = –4.46, *Z*_*Hippocampus*_ = –2.25) and *SCD* (cg26351966, *Z*_*Blood*_ = –4.86, *Z*_*Hippocampus*_ = –2.21) [51–53]. However, several VMPs exhibited opposite patterns of variance between blood and brain regions, including some within the *GRIK3*, *GFI1*, *ADRB3* and *MND1* genes, amongst others, indicating that some schizophrenia associated VMPs may exhibit divergent variance patterns between the periphery and brain. Finally, 2169 blood DMPs were also detected in the four tested brain regions, of which 1056 were concordant, 912 were discordant, and 201 exhibited both increased and decreased methylation across multiple regions in the brain (Table S17).

### Schizophrenia VMPs are underrepresented amongst genomic regulatory features

The genomic distribution of VMPs was additionally analysed to explore the impact of schizophrenia associated DNA methylation variance on gene expression and regulation. Utilising genomic features mapped by *PsychENCODE* [54], we identified significant (*FDR*_*BH*_ < 0.05) underrepresentation of VMPs within promoters (*OR* = 0.66), and H3K27ac peaks mapped in the cerebellar and temporal cortices (*OR* ≤ 0.46, Fisher’s Exact Test; Fig. 3c, Table S18). In addition, there was nominal evidence for underrepresentation of VMPs in H3K27ac peaks in the prefrontal cortex (*OR* = 0.65, *P* = 0.04), and overrepresentation within enhancers (*OR* ≥ 1.42, *P* = 0.03; Fig. 3c, Table S18). Using genic features from the Illumina 450K manifest, VMPs were also significantly underrepresented within transcription start sites (*OR* ≤ 0.53) and first exons (*OR* = 0.37), and overrepresented amongst gene bodies (*OR* = 1.41), while nominal overrepresentation was additionally identified amongst intergenic regions (*OR* = 1.43, *P* = 0.02; Fig. 3c, Table S18). These enrichment profiles were again most strongly observed amongst VMPs with increased variance in schizophrenia, whereas VMPs with decreased variance exhibited little evidence for enrichment or underrepresentation. Despite the limited concordance between variance and mean effects, enrichment profiles for DMPs were largely similar to VMPs, noting that DMPs were generally more-significant (Fig. 3c, Table S18). There was, however, divergence with respect to CpG islands, wherein VMPs exhibited limited enrichment, while DMPs were significantly overrepresented within shelves and open sea regions (*OR* ≥ 1.11), and underrepresented within islands and shores (*OR* ≤ 0.75; Fig. S10, Table S18).

### Variable methylation of known schizophrenia-associated genes

**Fig. 4:**
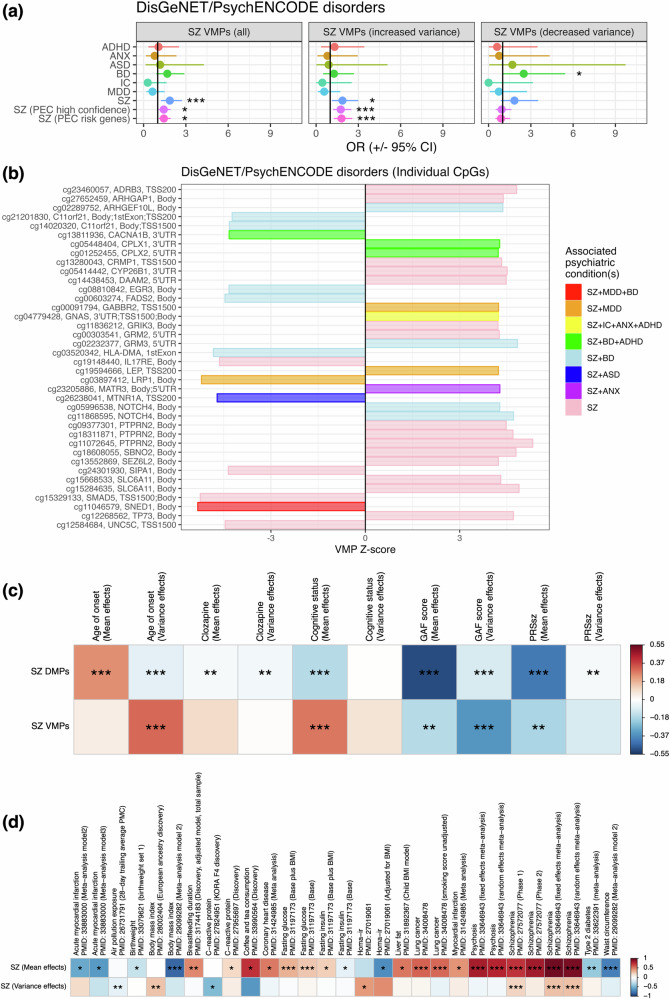
Genes previously associated with schizophrenia observed amongst VMP signatures. **a** Forest plot depicting enrichment of schizophrenia VMPs within genes associated with psychiatric conditions, as defined by *DisGeNET* [55] curated and inferred gene-disorder pairings, and *PsychENCODE* [54]. Data presented as odds ratios ± 95% confidence interval. * = *P* < 0.05, *** = *P*_*Bonf*_ < 0.05. ADHD = attention deficit hyperactive disorder, ANX = anxiety, ASD = autism spectrum disorders, BD = bipolar disorder, IC = impaired cognition, MDD = major depressive disorder, SZ = schizophrenia, PEC high confidence and risk gene sets denote schizophrenia associated genes from *PsychENCODE*. **b** Variance *Z*-scores for schizophrenia VMPs within genes previously associated with psychiatric illness. VMP ids (y-axis) are reported as CpG identifier, annotated gene, and genic position. **c** Heatmap presenting epigenetic covariance for schizophrenia VMPs and DMPs (y-axis) with respect to mean and variance effects for features of schizophrenia measured in the Australian Schizophrenia Research Bank (x-axis). Scale denotes Pearson’s correlation coefficient, with red = positive covariance and blue = negative covariance. * = *P* < 0.05, ** = *FDR*_*BH*_ < 0.05, *** = *P*_*Bonf*_ < 0.05. **d** As in **c**, except depicting covariance between CpGs associated with various traits and exposures reported in *EWASCatalog* [40] (*x*-axis) and their corresponding mean and variance effects in association with schizophrenia.

Gene-disorder associations from *DisGeNET* [55] and *PsychENCODE* were employed to determine whether schizophrenia VMPs were enriched amongst genes previously associated with psychiatric illness. Interestingly, there was evidence for significant (*FDR*_*BH*_ < 0.05) overrepresentation of schizophrenia VMPs amongst genes previously associated with this disorder in *DisGeNET* (*OR* = 1.86, Fisher’s Exact Test) and nominal evidence using *PsychENCODE* (*OR* ≥ 1.43, *P* ≤ 0.02), however, no other psychiatric conditions were identified (Fig. 4a, Table S19). Similarly, DMPs exhibited significant evidence for overrepresentation amongst *PsychENCODE* schizophrenia-associated gene sets (*OR* ≥ 1.39) and major depressive disorder (*OR* = 1.15), while anxiety was underrepresented (*OR* = 0.77; Fig. S11, Table S19). We subsequently analysed the 213 schizophrenia-associated VMPs individually for known gene-disorder annotations involving psychiatric conditions. Overall, 37 VMPs were associated with at least one psychiatric condition, noting schizophrenia was represented in all cases (Fig. 4b, Table S20). Amongst these, we identified a range of genes pertinent to neuronal function, including genes associated with neurotransmitter receptors: *ADRB3*, *GABBR2*, *GRIK3*, *GRM2*, *GRM3*, *MTNR1A*, ion channels: *CACNA1B* and *SLC6A11*; synaptic vesicle release: *CPLX1*, *CPLX2* and *PTPRN2*; axon guidance: *CRMP1* and *UNC5C*, and circulating biochemical factors: *CYP26B1*, *FADS2*, *LEP* and *LRP1*, amongst others, noting these broad functional categories are not mutually exclusive (Fig. 4b, Table S20). Furthermore, the single VMP associated with FEP was also associated with schizophrenia and major depressive disorder through the serine/threonine kinase *STK19* (Table S20).

### Epigenetic covariance between schizophrenia VMPs and measures of progression and severity

To extend these analyses, schizophrenia associated VMPs and DMPs were compared to their corresponding effect sizes in association with five features of schizophrenia, as measured within the Australian schizophrenia research bank (ASRB; see Supplementary Methods) [6]. Interestingly, at an *FDR*_*BH*_ < 0.05, schizophrenia VMPs were correlated with mean effects for cognitive deficits (*r* = 0.29), schizophrenia polygenic risk scores (*r* = –0.18) and Global Assessment of Function (GAF) scores (*r* = –0.16), suggesting VMPs for schizophrenia may also be associated with clinically relevant features of this disorder (Fig. 4c, Table S21). Stronger correlation was observed with variance effects for GAF scores (*r* = –0.35) and age of onset (*r* = 0.31), indicating potential overlap between DNA methylation variance for schizophrenia diagnosis and these measures. Analysis of schizophrenia DMPs additionally revealed extensive correlation with all tested measures of progression and severity after correction for multiple testing, except for cognitive status mean effects (Fig. 4c, Table S21).

### Phenome-wide epigenetic covariance

To further explore the functional significance of schizophrenia associated DNA methylation variance, EWAS mean effects for traits reported in *EWASCatalog* [40] were compared to their corresponding meta-analysis variance effects for schizophrenia. As anticipated, positive epigenetic covariance was uncovered between schizophrenia DNA methylation variance and three EWAS of schizophrenia at a Bonferroni-corrected *P* < 6.1 x 10^–6^, noting that two of these EWAS feature some of the cohorts included in the present study (Fig. 4d, Table S22). At an *FDR*_*BH*_ < 0.05, significant covariance was observed with respect to air pollution exposure (*r* = –0.11) and body mass index (BMI, *r* = 0.27), while C-reactive protein (*r* = –0.47) and homoeostatic model assessment of insulin resistance (HOMA-IR, *r* = 0.42) were nominally associated (Fig. 4d, Table S22). Many of these associations were also revealed by analysis of schizophrenia mean effects, wherein additional traits were uncovered such as acute myocardial infarction, birthweight, fasting glucose, lung cancer and psychosis (Fig. 4d, Table S20). Finally, we observed substantial covariance between schizophrenia associated DNA methylation and mean effects for plasma protein concentrations at an *FDR*_*BH*_ < 0.05, including 70 proteins associated with schizophrenia DNA methylation variance and 306 associated with mean effects (Table S22). In both cases, functional enrichment analysis converged on haemostasis and immune function (Table S23).

## Discussion

Epigenomic dysregulation is a well-established feature of psychiatric conditions [6–12], that has functional implications for individual-to-individual phenotypic heterogeneity and treatment response. In this study, we conducted the largest epigenome-wide analysis of DNA methylation variance in schizophrenia and FEP to explore epigenetic contributions to heterogeneity and function. Our analyses revealed a range of schizophrenia associated VMPs enriched within genes that are differentially expressed in the brain and/or associated with neuronal function. Strikingly, some VMPs also exhibited concordant changes in post-mortem brain tissue, suggesting a proportion of blood VMPs may serve as a surrogate marker for changes occurring in the brain. Interestingly, we identified genes associated with fatty acid metabolism amongst VMPs with concordant changes in blood and brain, including *HSD17B4*, *CYP4V2*, *FADS2*, and *SCD* [51–53], indicating altered DNA methylation variance may impact fatty acid metabolism in these tissues in schizophrenia. However, we note these genes were subject to decreased variance, the functional impact of which is yet to be elucidated. These findings are nonetheless broadly consistent with observational studies reporting altered lipid metabolism and concentrations in the prefrontal cortex [56–59] and serum [60, 61] of affected individuals, and recent evidence suggesting omega-3 supplementation may improve symptom severity in deficient individuals [62]. We also identified concordant blood and brain VMPs within *SBNO2*, a regulator of IL-6 signalling associated with increased neuroinflammation and neurodegeneration in murine knockout models [46], and *DOC2B*, a calcium sensor associated with spontaneous neurotransmitter release [47, 48, 50]. While it is unclear if these VMPs also contribute to phenotypic heterogeneity in schizophrenia, their functional associations with inflammation and neurotransmission broadly support a role in this disorder [63–66].

Large disparity was observed between the number of loci associated with schizophrenia (213 VMPs, 9389 DMPs) and FEP (1 VMP, 271 DMPs), potentially arising from divergent statistical power and/or (epi)genetic architecture between these traits. Interestingly, a meta-analysis combining schizophrenia and FEP (*n*_*Controls*_ = 1387, *n*_*Psychosis*_ = 1680) only identified 31 VMPs, including 29 that were also identified in the schizophrenia meta-analysis, and two novel VMPs affecting genes associated with neurodevelopment (*TBC1D19*) and dendritic spine density (*AHRR*) in schizophrenia [67, 68] (Figs. S12–S18, Tables S24–S29). This increased statistical power may therefore be offset by divergent (epi)genetic architecture, which may arise from distinct diagnostic criteria between FEP (acute psychosis that may progress to full recovery or chronic psychosocial impairment [69]) and schizophrenia (chronic mental illness [70]). However, further investigation with greater sample size is required to confirm these findings. Nonetheless, we note that evidence for positive correlation between schizophrenia and FEP methylation profiles in the current study suggests these traits may share some epigenetic architecture that could yield clinical utility together with genetic risk [71, 72]. Similarly, we also conducted sex-specific meta-analyses considering the known sexual dimorphism in these disorders [73–75]. Strikingly, significant DMPs were only identified for males across all traits, consistent with previously observed sex biases for schizophrenia-associated gene expression [73, 74], and furthermore, few sex-specific VMPs were uncovered, potentially owing to decreased statistical power (Table S30).

Interestingly, significant covariance was identified between schizophrenia VMPs and their corresponding effect sizes for measures of progression and severity, such as age of onset, cognitive deficits, and GAF scores. This finding is particularly exciting given that VMPs associated with schizophrenia diagnosis may therefore index specific features of the disorder and could potentially yield classification-based utility in future studies using methods such as epigenetic scoring [6, 18, 19]. Notably, we report 44 specific VMPs that also exhibited nominal mean effects in association with measures of progression and severity that may guide future work (Table S31). Phenome-wide analyses also uncovered evidence for epigenetic covariance between schizophrenia DNA methylation variance and risk-altering factors including air pollution exposure [76, 77], BMI [78, 79], C-reactive protein [80–83] and insulin resistance [84, 85], raising the possibility that some schizophrenia-associated DNA methylation variance is interrelated with known correlates of this disorder. Strikingly, growing evidence suggests variable methylation captures lifetime exposure to environmental factors and stochastic changes associated with aging that may also contribute to schizophrenia aetiology, progression, and heterogeneity [15, 86–88]. It is also plausible that some DNA methylation variance may index common genetic variants associated with psychiatric illness or other risk factors [89]. Indeed, we investigated this by identifying known methylation quantitative trait loci for VMPs in the current study and assessing their phenotypic associations, identifying a vast range of traits including diseases, neuroimaging phenotypes, biochemical measures, expression/protein quantitative trait loci and other quantitative traits and phenotypes (Fig. S19, Tables S32, S33). These findings indicate that some common variants associated with VMPs in schizophrenia are also significantly associated with a broad range of traits, however additional investigation is required to ascertain these results.

Substantial divergence was also observed between schizophrenia-associated DNA methylation variance and mean effects, emphasised by the detection of only 10 CpGs exhibiting evidence for both variable and differential methylation. Interestingly, this is consistent with mounting evidence suggesting VMPs represent their own class of DNA methylation changes that tend to partition from mean effects, particularly in the context of aging [15, 86]. It remains unclear, however, if DMPs and VMPs impact gene expression via shared or distinct mechanisms. In the present study, genomic enrichment profiles were largely consistent between VMPs and DMPs, characterised by underrepresentation amongst H3K27ac peaks, promoters, transcription start sites and first exons. While DNA methylation within these regions is thought to impact *cis*-acting transcriptional activity [13, 14], methylation events outside of these regions is poorly understood, thus it is difficult to speculate the mechanistic basis by which these DMPs and VMPs broadly impact gene expression. Indeed, the functional significance of variable methylation may be most pertinent on a gene-by-gene basis. However, there were also varying degrees of evidence supporting DMP and VMP enrichment within intergenic sequences and gene bodies, suggesting these CpGs may, respectively, be important for *trans*-acting regulation of gene expression, or modulation of cotranscriptional splicing and cryptic promoters [13]. Nonetheless, we suspect that similar genomic distributions indicate that schizophrenia associated VMPs and DMPs may converge on similar regulatory systems despite involving distinct CpGs. We also acknowledge that some VMPs exhibited decreased variance in schizophrenia, which may reflect CpGs that become less responsive to genetic or environmental factors in individuals with this disorder [15].

In conclusion, our results suggest schizophrenia is associated with variably methylated genomic loci which may contribute to heterogeneity in this disorder, however, we acknowledge several caveats when interpreting these findings. Firstly, our findings require validation in larger, independent cohorts, particularly since large sample sizes are required to reliably detect VMPs versus DMPs [15]. This is particularly relevant for the analysis of brain methylation profiles, wherein sample sizes were particularly low, owing to the limited availability of post-mortem brain samples. Additional validation is also necessary to explore the suitability of blood as a surrogate for brain DNA methylation in schizophrenia, given that recent studies report varying patterns of correlation between these tissues [90–93], and in the present study, many blood VMPs exhibited no or opposite changes in the analysed brain regions, including key neuronal genes such as *GRIK3*, *GFI1*, *ADRB3* and *MND1*. Similarly, while there was minimal evidence for changes in cell-type proportion variance between cases and controls in the present study (Fig. S20, Table S34), future work may benefit from cell-type deconvolution, noting existing methods for mean effects [94] cannot be directly applied to DNA methylation variance, and larger sample sizes are likely required. Furthermore, our selection of covariates was not exhaustive, since we were limited to publicly available phenotype data and methylation derived measures, thus factors such as antipsychotic medications could not be broadly accounted for. However, we note the covariates utilised in the current study are largely consistent with recent EWAS of schizophrenia and related phenotypes [6, 7, 9–12]. Finally, this study utilised 450 K and EPIC arrays that sample 1.6–3% of the methylome, therefore some important VMPs may remain undetected [95]. Whole genome bisulphite sequencing could yield VMPs with greater resolution [96] in future studies, however we acknowledge the substantial cost increase versus array-based methods, and the limited abundance and power of existing public data [97]. Despite these caveats, our findings indicate that epigenetic variance represents an exciting prospect for disentangling molecular heterogeneity in schizophrenia, with a view of reconciling molecular and phenotypic heterogeneity and informing personalised interventions.

## Supplementary information

Supplementary methods and results

Supplementary tables

## Data Availability

Raw signal intensities and detection *P*-values were obtained from the Gene Expression Omnibus as follows: UCL: GSE84727, DUB: GSE147221, IoPPN: GSE152027, EU-GEI: GSE152026, cerebellum: GSE89702, hippocampus: GSE89703, prefrontal cortex: GSE74193 and striatum: GSE89705 and GSE89706. EWAS summary statistics containing mean and variance effects for these cohorts and the ASRB are available at https://drive.google.com/drive/folders/1VWYbe35IJO8VDDTO3P7zqsm6rmUFxTv4?usp=share_link. Public access to individual methylation and phenotype data are restricted by the collection human ethics and the conditions of the material transfer agreement. Interested parties are encouraged to contact the corresponding author regarding collaborative data sharing.

## Code availability

Code for array processing, within-cohort EWAS and meta-analyses are available at: https://github.com/dkiltschewskij/Schizophrenia_DNAm_Variance/.
