# Supplementary material for: Schizophrenia is associated with altered DNA methylation variance
Source: Mol Psychiatry. 2024 Sep 13;30(4):1383–95. doi: 10.1038/s41380-024-02749-5 (PMC11919772; doi:10.1038/s41380-024-02749-5)
Supplement: Supplementary file 1 — Supplementary methods and results [file 41380_2024_2749_MOESM1_ESM.docx]

**SUPPLEMENTARY INFORMATION**

**Schizophrenia is Associated with Altered DNA Methylation Variance**

Kiltschewskij *et al*.

**SUPPLEMENTARY METHODS**

**Differentially and variably methylated regions**

Differentially and variably methylated regions (DMRs and VMRs) were identified using *combp* [1], as implemented in the *ENmix R* package (v1.25.1) [2]. For each probe, meta-analysis *P*-values and genomic positions were used as input, employing a maximum window size of 1kb, autocorrelation bin size of 50bp and seed *FDR* of 0.05. Regions with *P_Sidak_* < 0.05 and ≥ 2 probes were considered DMRs/VMRs.

**Functional enrichment**

The *gProfiler2* (v.0.2.2) [3] functional enrichment analysis tool was utilised to examine enriched pathways (*KEGG*, *Reactome* and *WikiPathways*), gene ontology (GO) terms, proteins (*Human Protein Atlas* and *Comprehensive Resource of Mammalian Protein Complexes*) and microRNA binding sites (*miRTarBase*) amongst genes associated with VMPs or DMPs, with *FDR_BH_* < 0.05 considered significant. These genes were also examined for enrichment amongst tissue gene expression data sets from *GTEx* (v7) using the *deTS* *R* library (v1.0) [4] with default parameters, with *FDR_BH_* < 0.05 considered significant. VMP/DMP overrepresentation within genomic features was determined relative to the remainder of the epigenome via Fishers Exact Test, using enhancers, H3K27ac peaks and promoters from *PsychENCODE* [5], and transcription start sites, gene bodies, untranslated regions, intergenic sequences and CpG islands from *UCSC* [6].

**Analysis of DNA methylation associated with clinical features of schizophrenia**

EWAS were also conducted using 381 individuals with schizophrenia from the Australian Schizophrenia Research Bank (ASRB). Briefly, DNA methylation from peripheral blood mononuclear cells (PBMCs) was previously quantified via Illumina Infinium HumanMethylationEPIC BeadChip and subjected to processing and quality control as outlined in [7]. EWAS examining variance and mean effects were conducted in association with: age of onset, cognitive deficits, clozapine administration, global assessment of function (GAF) scores and common variant polygenic risk scores (PRS) for schizophrenia. Further details pertaining these phenotypes can be accessed at [7]. Levene’s Test was used as the primary test for binary traits (i.e. cognitive deficits and clozapine administration), while the Breusch-Pagan Test was employed to examine heteroskedasticity for continuous traits (i.e. age of onset, GAF scores and PRS) [8].

**Phenome-wide association studies of methylation quantitative trait loci associated with VMPs**

Methylation quantitative trait loci (mQTLs) for all schizophrenia associated VMPs were obtained from *GoDMC* (v0.1.0) [9]. Briefly, we retained 77 *cis*-acting (≤ 1Mb between the mQTL and CpG site), genome-wide significant (*P* < 5x10^–8^) mQTLs that survived linkage disequilibrium (LD) clumping and exhibited low heterogeneity (*P_Het_* > 0.05). Of these, we excluded eight insertions/deletions (INDELS) and three single nucleotide variants (SNVs) residing within the major histocompatibility (MHC) region (chr6:28477797-33448354), to ensure our downstream analyses were not biased by the complex LD patterns in this region. For the remaining mQTLs, we conducted phenome-wide association studies (pheWAS) across 50,037 binary and quantitative traits within the *IEUGWAS* database (v7.5.20) [10, 11], as well as 2,272 binary disease/disorder traits from *FinnGen* (release 9) [12]. For each analysis, *P*-values were corrected across all mQTLs and traits using the Bonferroni and Benjamini-Hochberg methods. While these analyses were attempted for the single FEP-associated VMP which surpassed correction for multiple testing, this VMP was found to reside within the MHC region, precluding further analysis.

**SUPPLEMENTARY FIGURES**

**
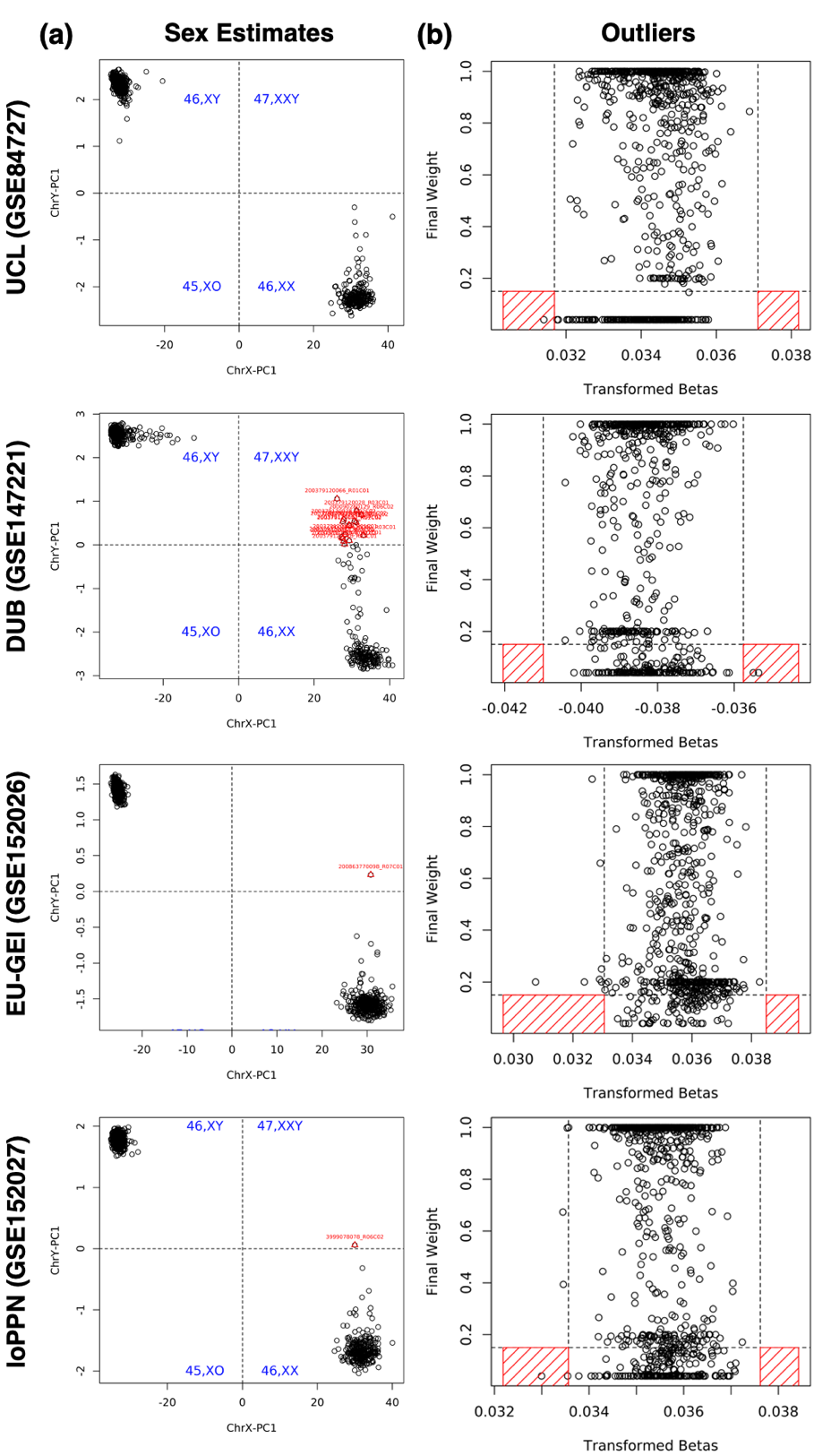
**

**Figure S1. Methylation-derived sex estimates and detection of outliers. (a)** Estimated sex for the four cohorts utilised in the primary meta-analyses of schizophrenia and first episode psychosis, as determined via the *wateRmelon* [13] (v1.35.2) *R* package. Note the detection of individuals with a predicted XXY genotype, which were subsequently excluded from further analyses. **(b)** Identification of outlier samples using the *outlyx* command of the *wateRmelon* package. Red shaded regions denote outlier samples, wherein both the first principal component derived from analysis of sample-level beta values (prior to normalisation) was greater than two interquartile ranges from the median, and final weight derived from Mahalanobis distances was less than 0.15. These outliers were excluded from downstream analyses.

**
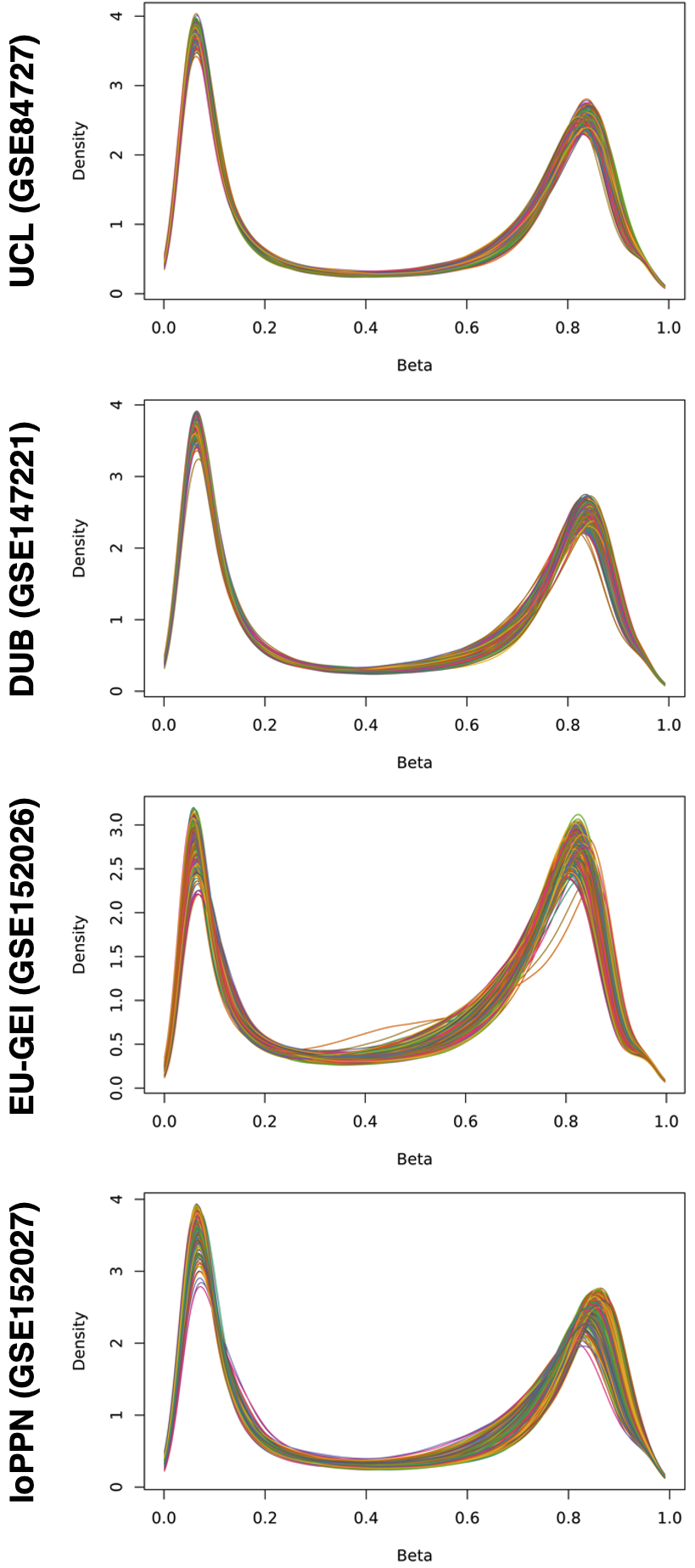
**

**Figure S2. Beta distributions after data processing and normalisation.** Beta distributions across all samples and cohorts included in the primary meta-analyses after data processing and normalisation. Since all samples followed the expected beta distributions, no samples were excluded.

**
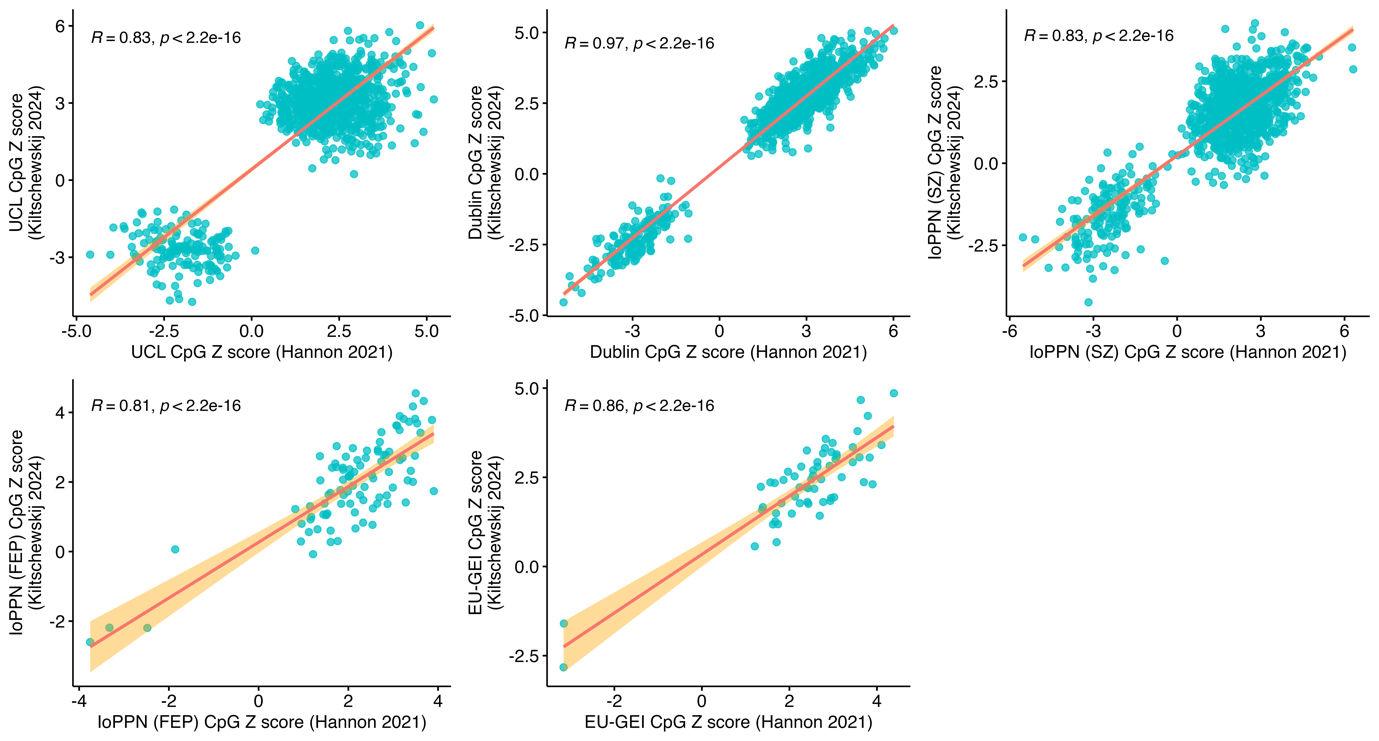
Figure S3. Univariate correlation between DMP effect sizes in the current study and Hannon et al 2021.** Comparison of Bacon-adjusted mean effects *Z*-scores (*β* / *SE*) between the present study and [14]. A total of 1,038 schizophrenia-associated probes and 95 FEP-associated probes were utilised as previously reported [14]. Note the strong correlation across all cohorts, suggesting our data processing and mean effects analyses were largely consistent with previous analyses.


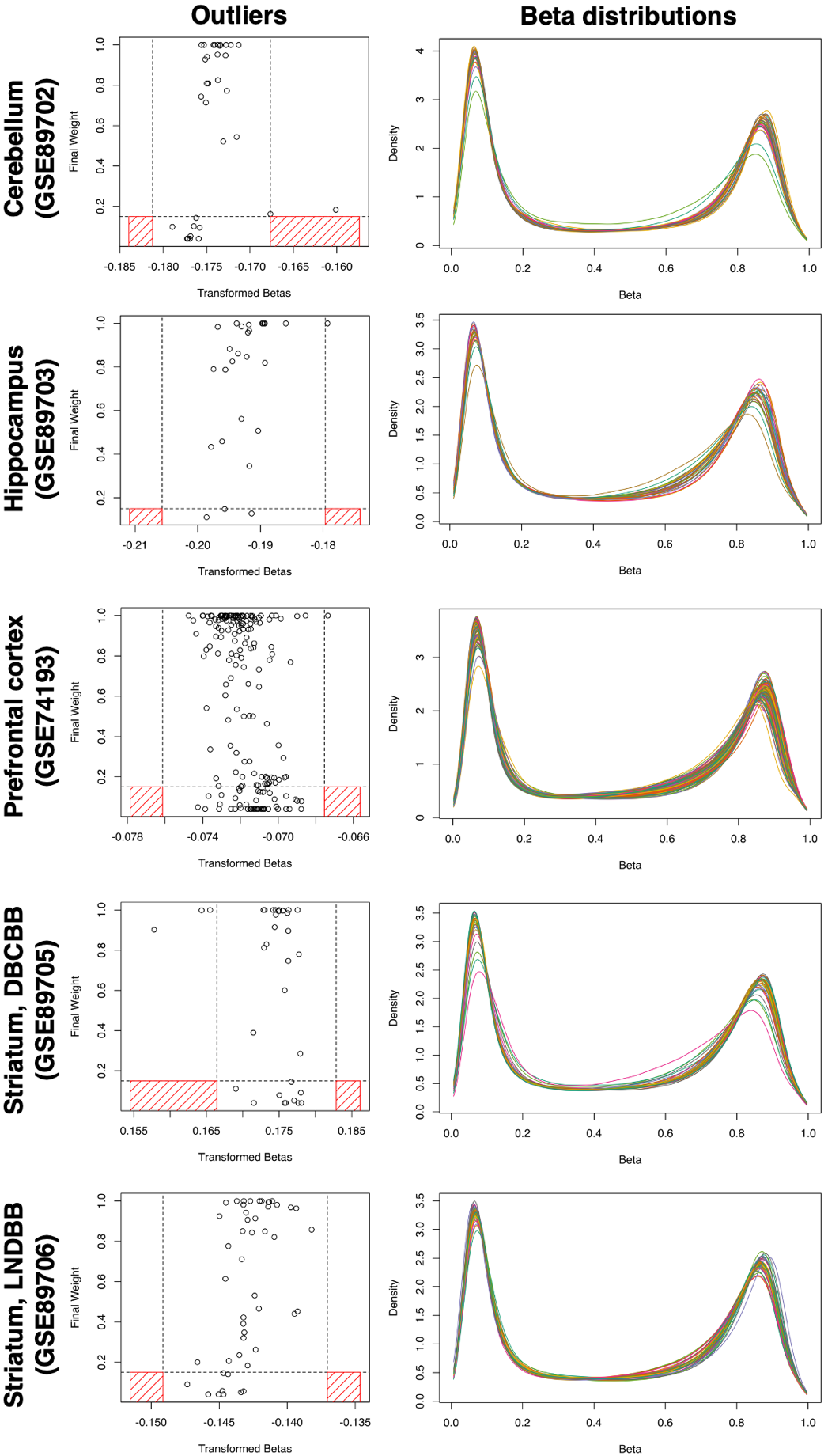


**(b)**

**(a)**

**Figure S4. Outliers and beta distributions from brain methylation data sets. (a & b)** As in **Fig. S1a** and **Fig. S2**, respectively, except depicting outliers **(a)** and beta distributions **(b)** for the five publicly available cohorts utilised for analysis of schizophrenia-associated DNA methylation variance and mean effects in post-mortem brain samples.

**
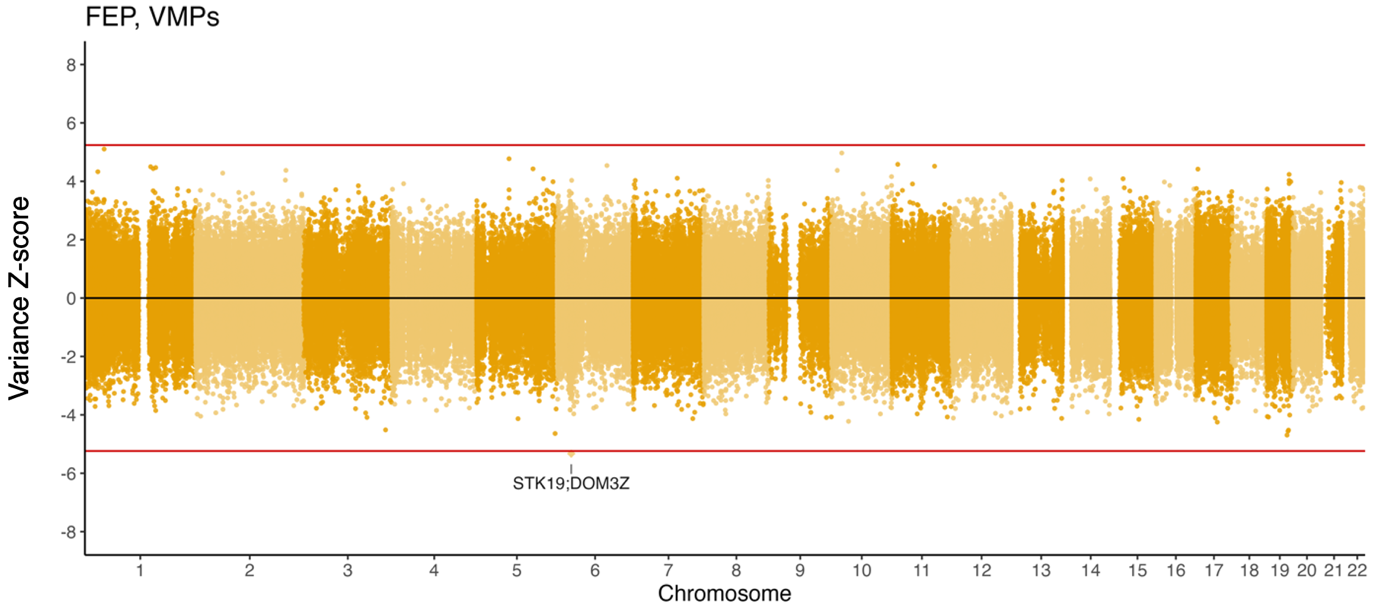
**

**Figure S5. Meta-analysis of DNA methylation variance in association with FEP.** Miami plot depicting the epigenome-wide distribution of meta-analysis *Z*-scores for DNA methylation variance in association with FEP. Positive *Z*-scores denote sites with increased variance in FEP, while negative *Z*-scores denote sites with decreased variance. Solid red line indicates the threshold for epigenome-wide significance (*P* < 1.61x10­^–7^­). The Benjamini-Hochberg FDR is not depicted in this figure, as this threshold was similar to the epigenome-wide significance threshold for this meta-analysis.


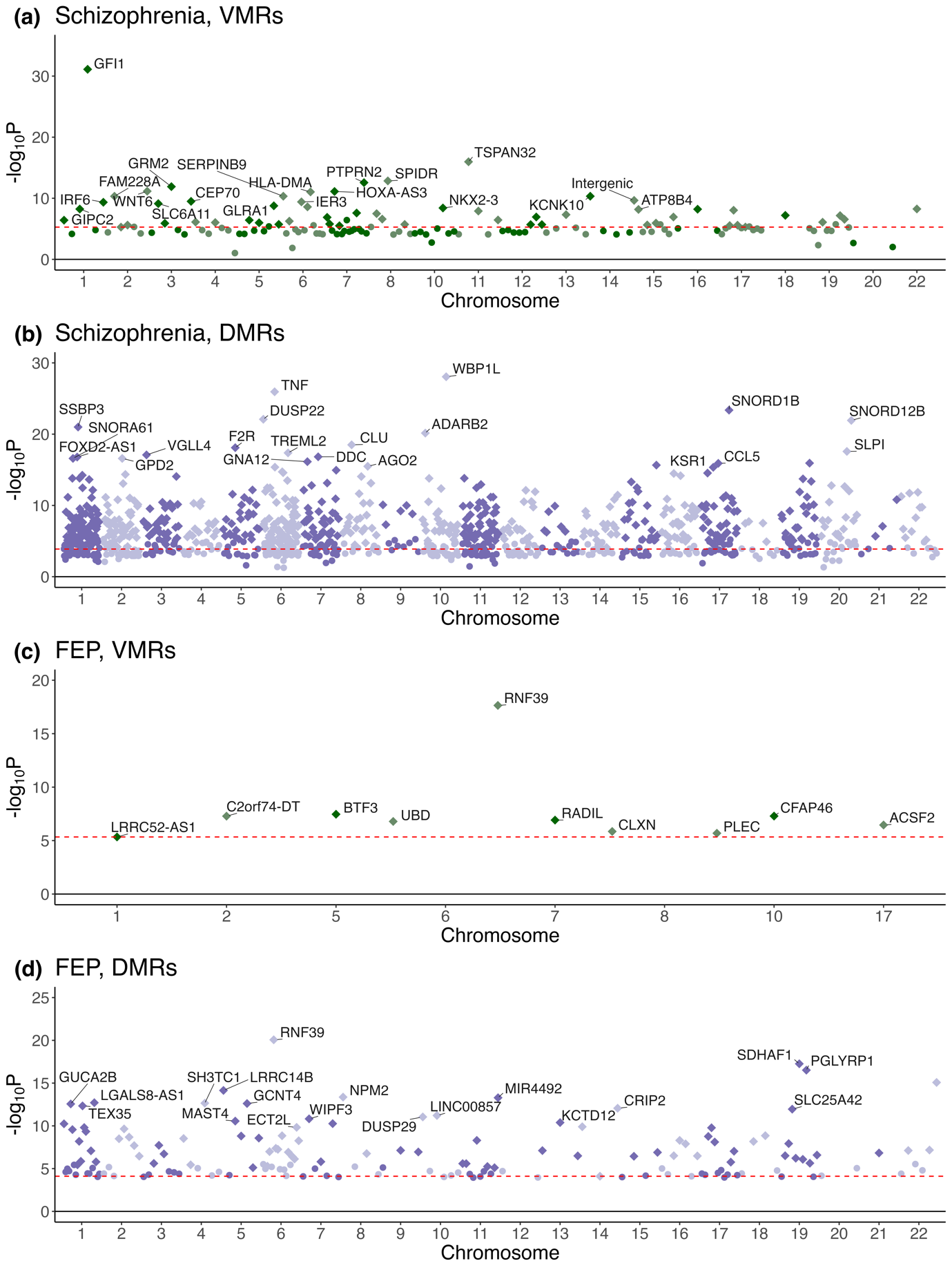


**Figure S6. Variably and differentially methylated regions. (a & b)** Genome-wide distribution of variably **(a)** and differentially **(b)** methylated regions associated with schizophrenia as identified via *combp*. The x-axis denotes chromosomal position and the y-axis denotes the –log_10_*P*-value. Horizontal red line corresponds to a Sidak-corrected *P_Sidak_* < 0.05, noting that some regions surpassed this threshold yet did not survive Sidak correction (circles), as this adjustment incorporates the DMR’s/VMR’s size. **(c & d)** As in **(a & b)**, except presenting variably **(c)** and differentially **(d)** methylated regions associated with FEP.

**
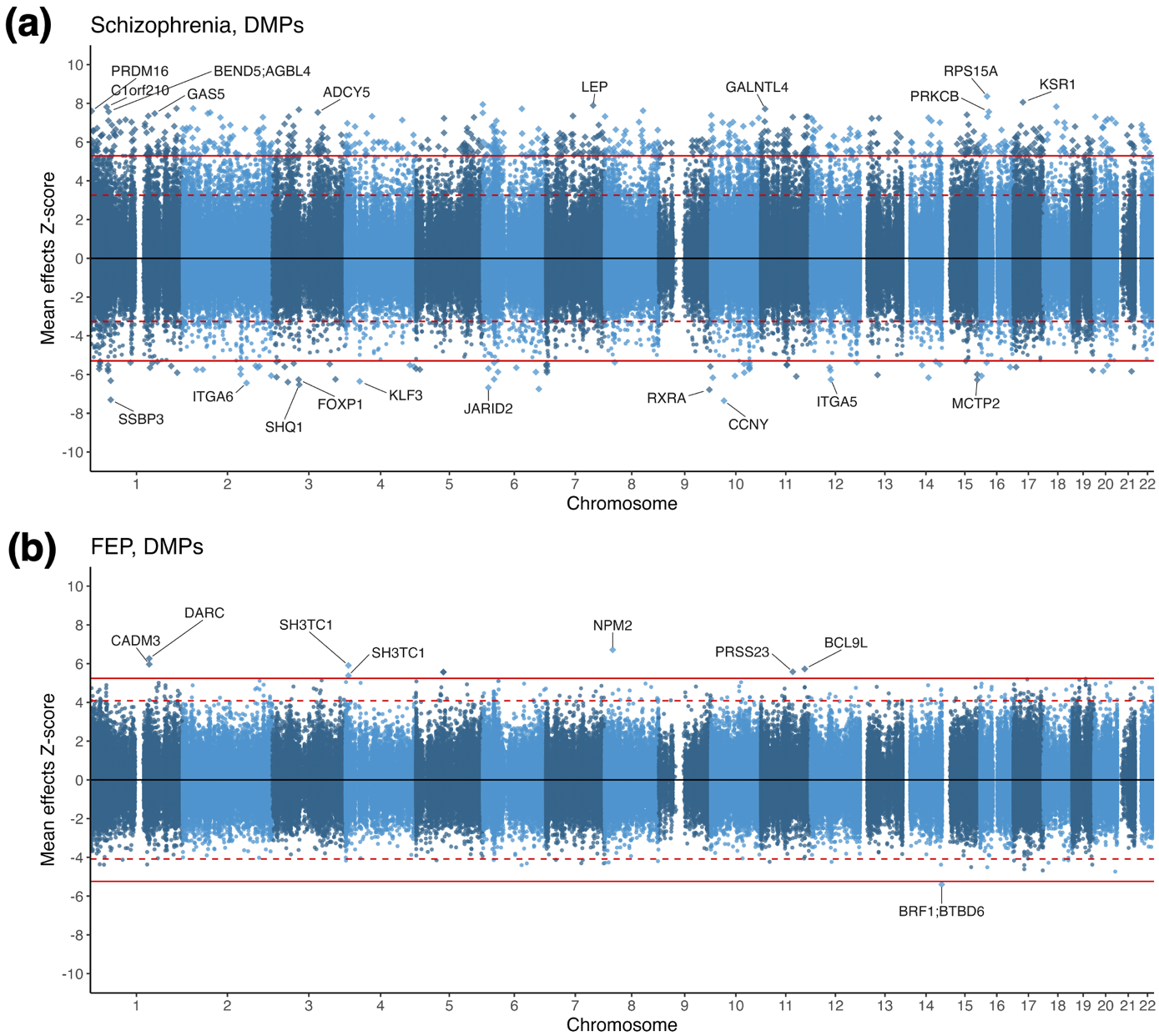
**

**Figure S7. Meta-analysis of DNA methylation mean effects.** Miami plots presenting epigenome-wide mean effects associated with **(a)** schizophrenia and **(b)** FEP. Positive *Z*-scores (*β* / *SE*) denote hypermethylated sites associated with each condition, while negative *Z*-scores denote hypomethylated sites. Solid red line indicates the threshold for epigenome-wide significance (*P_SZ_* < 1.2x10­^–7^, *P_FEP_* < 1.61x10­^–7^­­). Dashed red line indicates the Benjamini-Hochberg FDR.

**
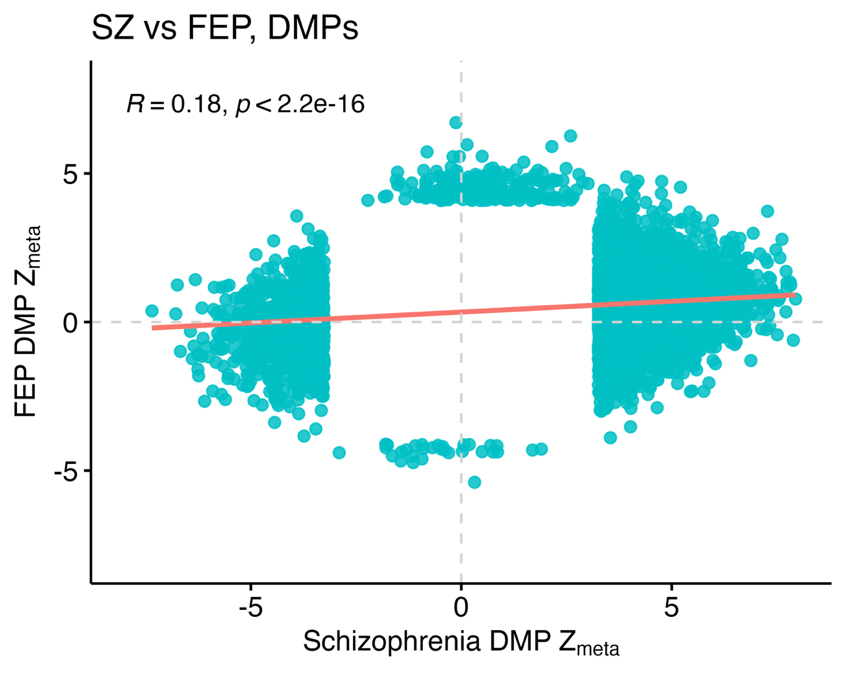
**

**Figure S8. Comparison of DMP *Z*-scores between the schizophrenia and FEP mean effects meta-analyses.** Scatter plot depicting *Z*-scores for all schizophrenia and/or FEP associated DMPs, revealing evidence for positive correlation of DNA methylation mean effects between both phenotypes. Pearson *r* and associated *P*-value reported top left.

**
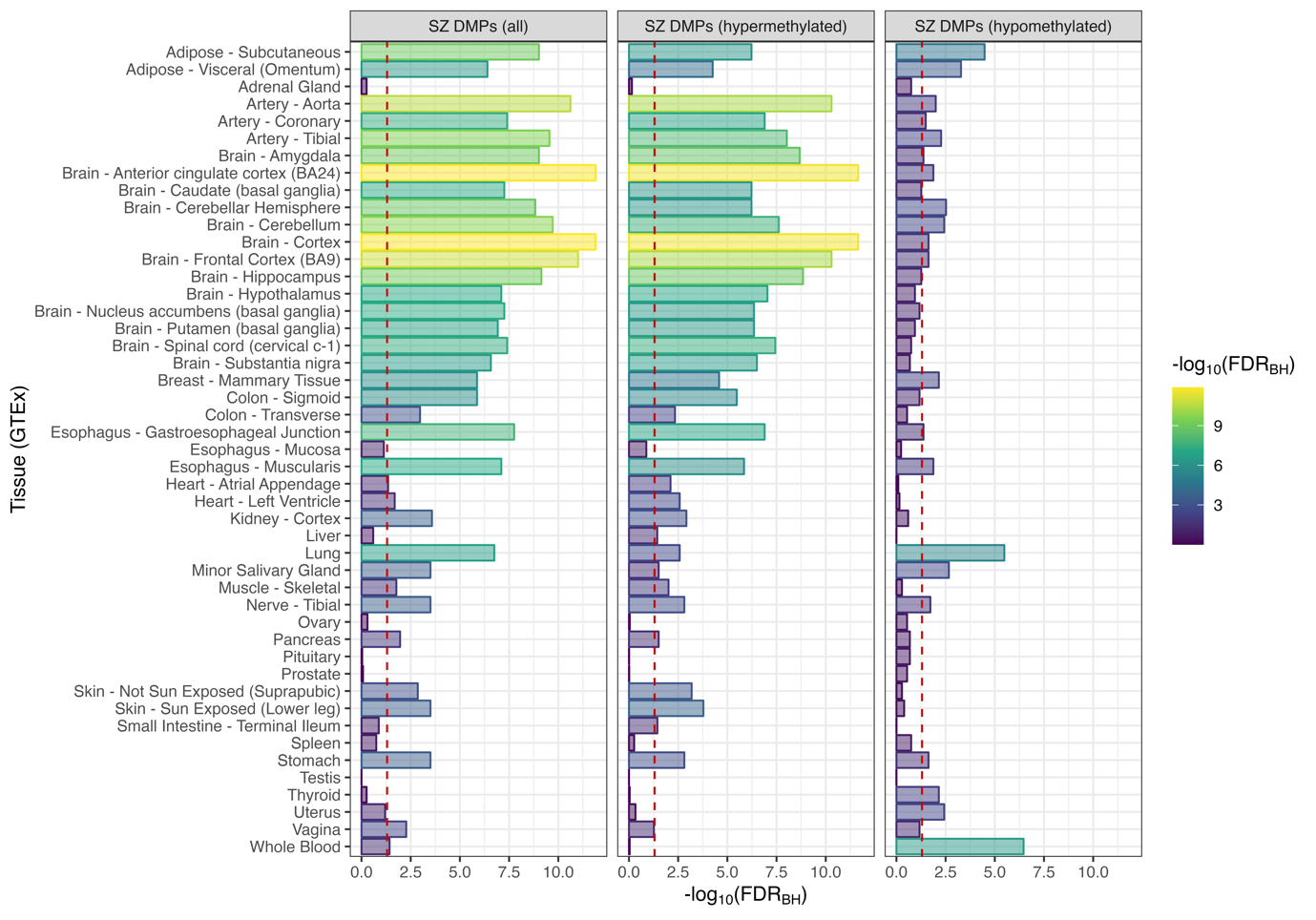
**

**Figure S9. Tissue enrichment profiles for schizophrenia associated DMPs.** Enrichment of schizophrenia associated DMPs amongst tissue gene expression profiles from GTEx (v7). For each tissue, genes expressed in the top 5% of all genes were deemed tissue-enriched. Vertical red line denotes *FDR_BH_ <* 0.05, as determined via Fisher’s Exact Test.

**
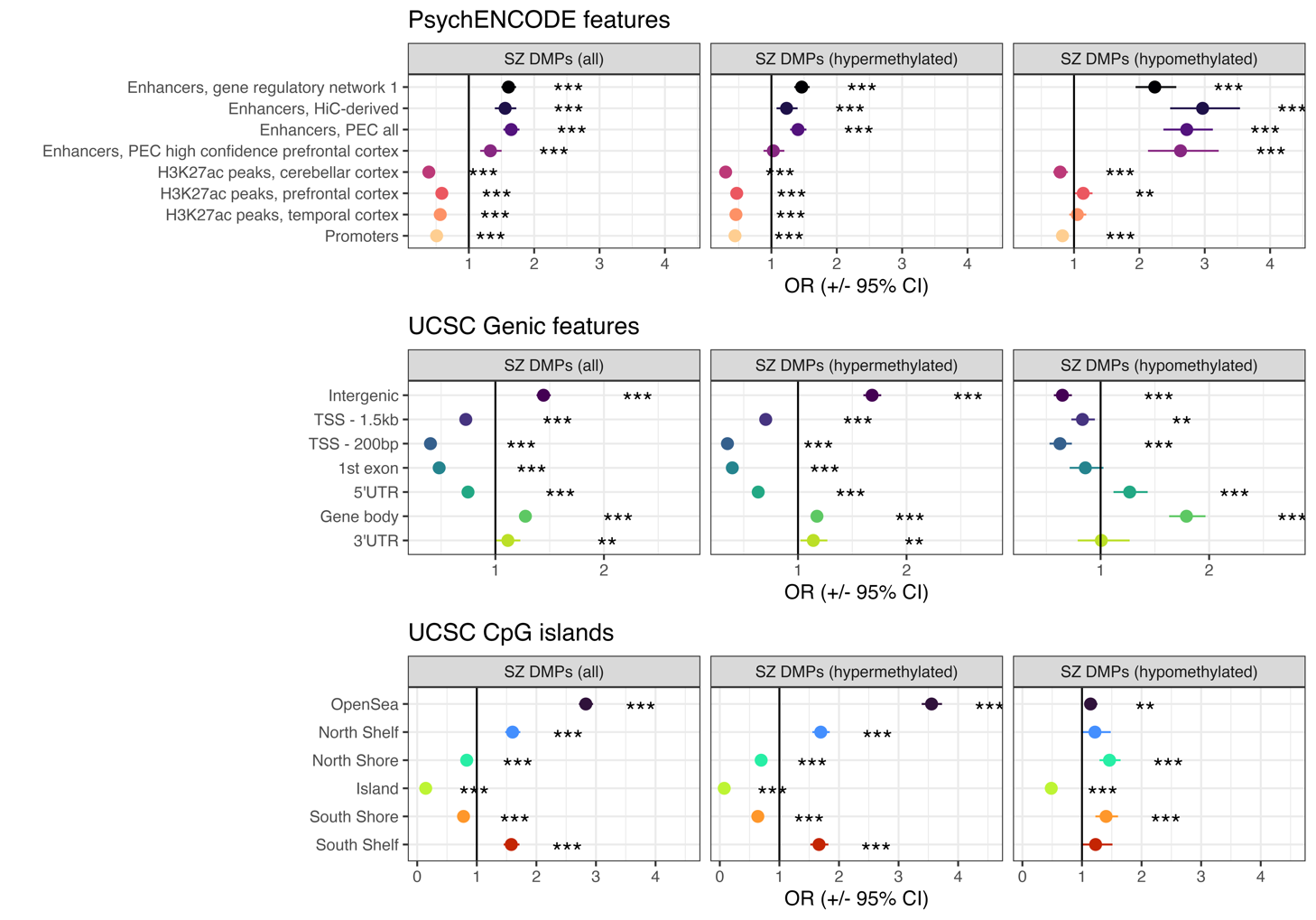
Figure S10. Genomic distribution of schizophrenia associated DMPs.** Forest plots depicting enrichment of schizophrenia associated DMPs within genomic regulatory regions reported by *PsychENCODE* [5], gene-centric features from UCSC [6] and CpG islands and neighbouring regions from UCSC. Data presented as odds ratios ± 95% confidence interval. * = *P* < 0.05, ** = *FDR_BH_* < 0.05, *** = *P_Bonf_* < 0.05.

**
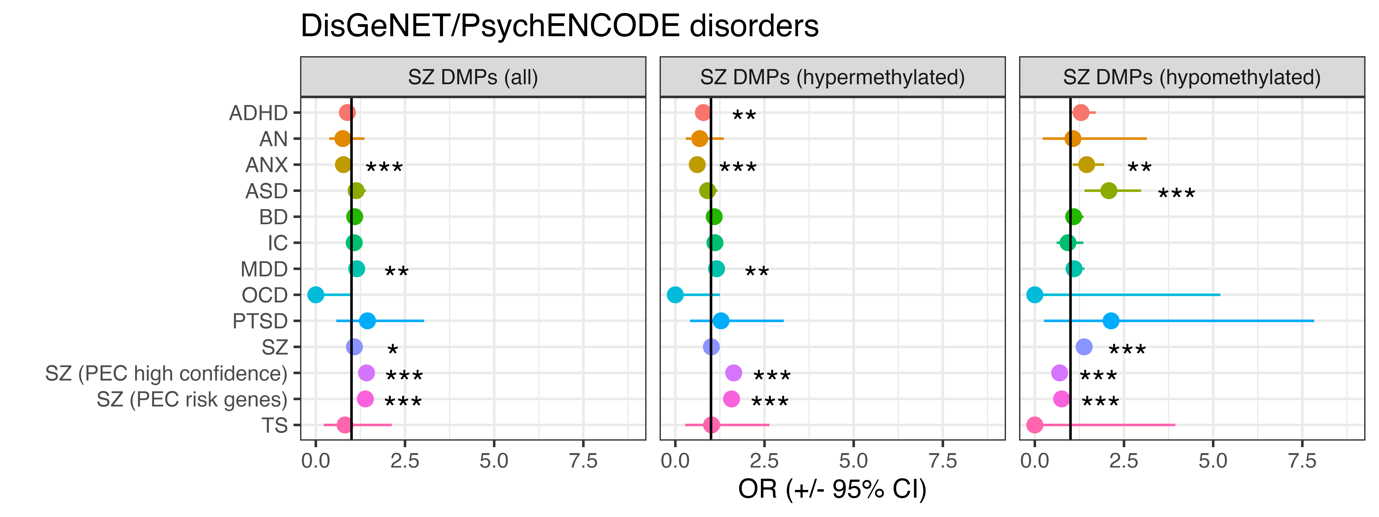
**

**Figure S11. Enrichment of schizophrenia associated DMPs within genes previously associated with psychiatric illness.** Enrichment of schizophrenia DMPs amongst genes associated with psychiatric conditions, as defined by *DisGeNET* [15] curated and inferred gene-disorder pairings, and *PsychENCODE* [5]. Data presented as odds ratios ± 95% confidence interval. * = *P* < 0.05, ** = *FDR_BH_* < 0.05, *** = *P_Bonf_* < 0.05. ADHD = attention deficit hyperactive disorder, AN = anorexia nervosa, ANX = anxiety, ASD = autism spectrum disorders, BD = bipolar disorder, IC = impaired cognition, MDD = major depressive disorder, OCD = obsessive compulsive disorder, PTSD = post-traumatic stress disorder, SZ = schizophrenia, TS = Tourette syndrome, PEC high confidence and risk gene sets denote schizophrenia associated genes from *PsychENCODE*.

**
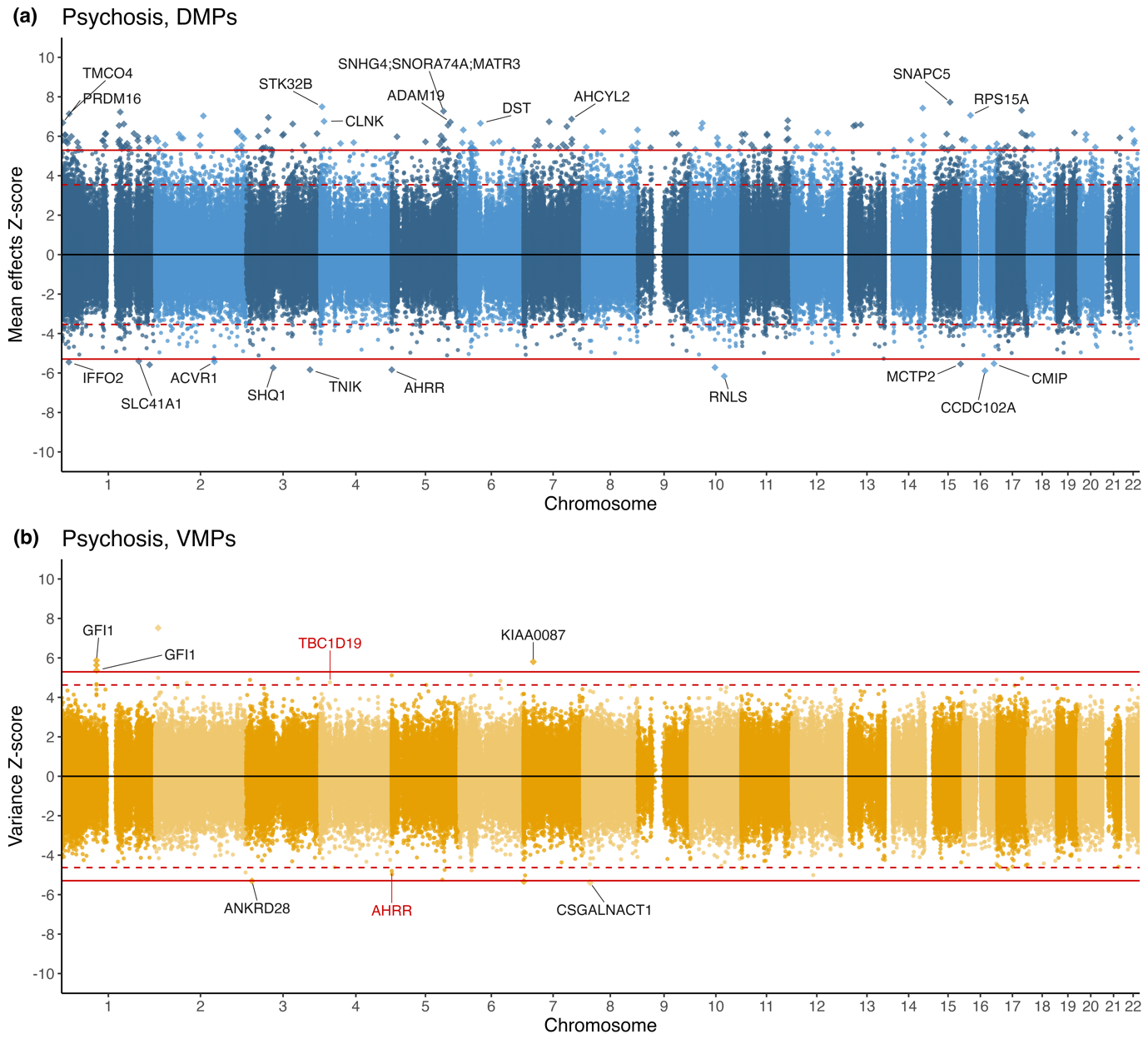
**

**Figure S12. Meta-analysis of DNA methylation mean effects and variance in association with psychosis. (a)** Miami plots presenting epigenome-wide mean effects associated with psychosis, obtained by combining schizophrenia and FEP diagnoses across the four cohorts used in the primary meta-analyses (*n_Controls_* = 1,387, *n_Psychosis_* = 1,680). Positive *Z*-scores (*β* / *SE*) denote hypermethylated sites associated with each condition, while negative *Z*-scores denote hypomethylated sites. Solid red line indicates the threshold for epigenome-wide significance (*P* < 1.2x10­^–7^). Dashed red line indicates the Benjamini-Hochberg FDR. **(b)** As in **(a)**, except analysing DNA methylation variance. Positive *Z*-scores denote sites with increased variance in psychosis, while negative *Z*-scores denote sites with decreased variance. Red labels denote novel VMP associations not detected in the primary meta-analyses of schizophrenia and FEP.

**
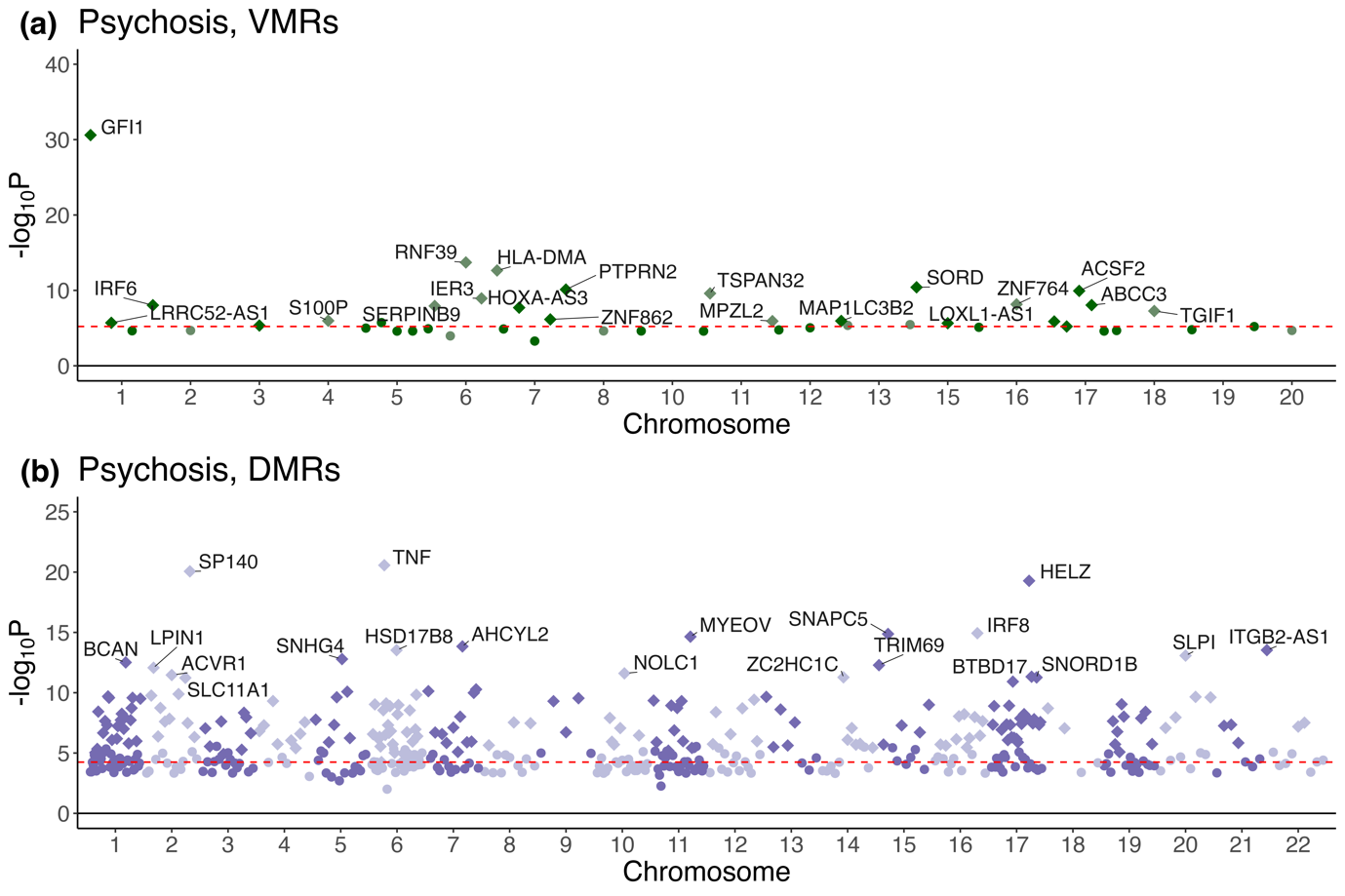
**

**Figure S13. Variably and differentially methylated regions associated with psychosis. (a & b)** Genome-wide distribution of variably **(a)** and differentially **(b)** methylated regions associated with schizophrenia as identified via *combp*. The x-axis denotes chromosomal position and the y-axis denotes the –log_10_*P*-value. Horizontal red line corresponds to a Sidak-corrected *P_Sidak_* < 0.05, noting that some regions surpassed this threshold yet did not survive Sidak correction (circles), as this adjustment incorporates the DMR’s/VMR’s size.

**
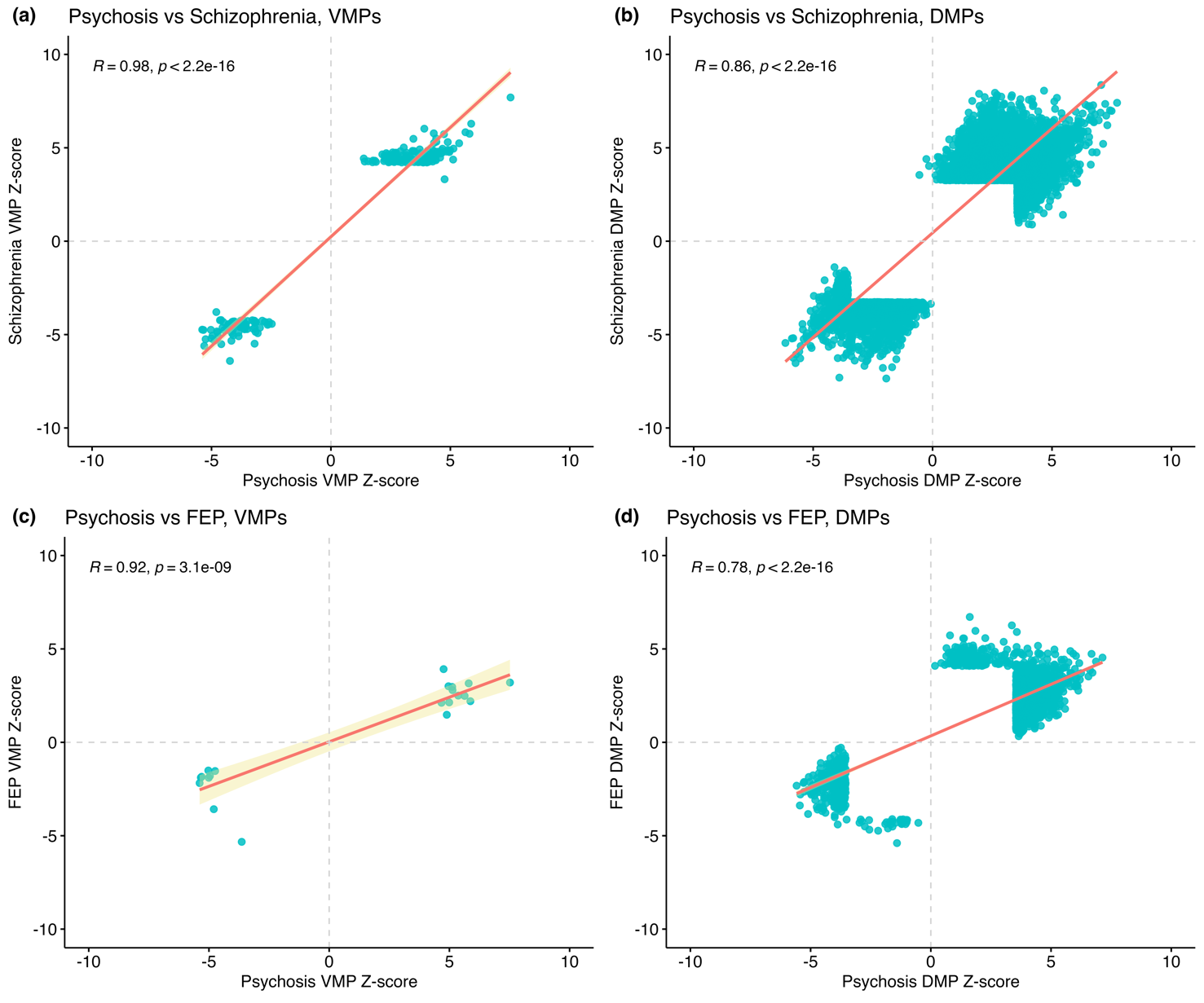
**

**Figure S14. Comparison of VMP and DMP signatures between psychosis, schizophrenia and FEP. (a)** Comparison of meta-analysis *Z*-scores for all VMPs significantly associated with psychosis and/or schizophrenia. Pearson correlation coefficient and associated *P*-value reported top left. **(b)** As in **(a)**, except comparing significant DMPs. **(c & d)** As in **(a & b)**, except comparing psychosis with FEP.


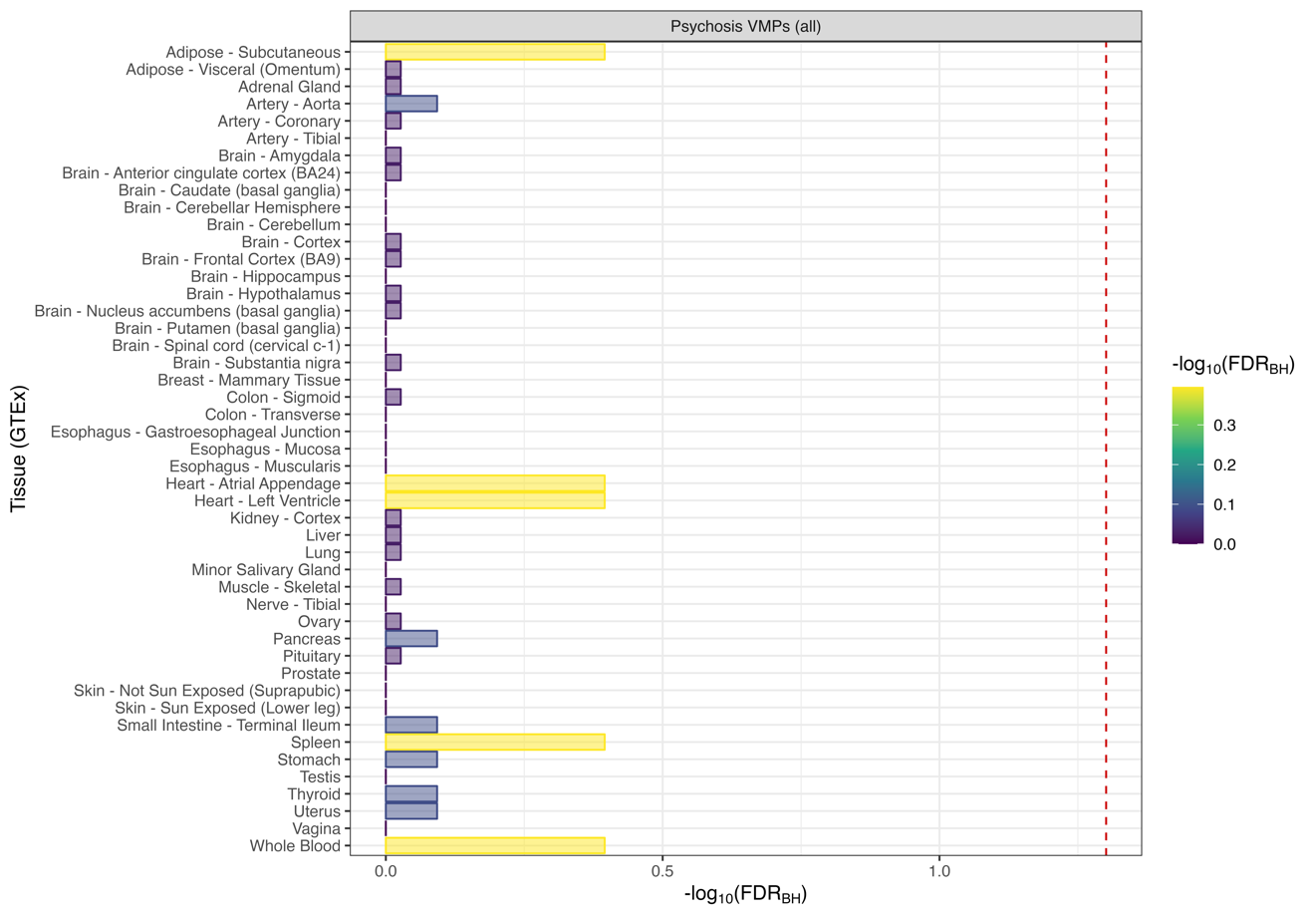


**(a)**

**(b)**


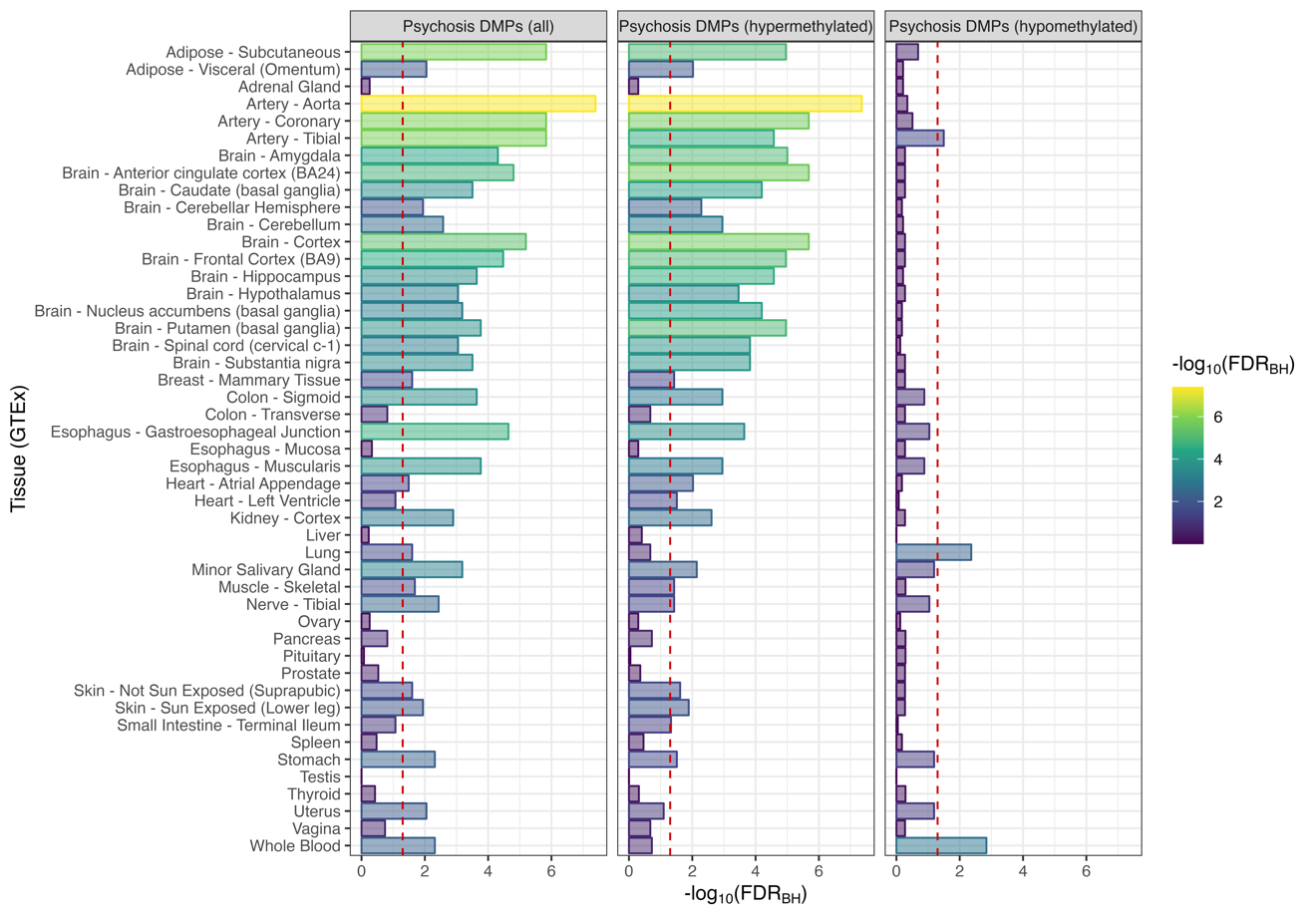


**Figure S15. Tissue enrichment profiles for psychosis associated VMPs and DMPs. (a)** Enrichment of psychosis associated VMPs amongst tissue gene expression profiles from GTEx (v7). For each tissue, genes expressed in the top 5% of all genes were deemed tissue-enriched. Vertical red line denotes *FDR_BH_ <* 0.05, as determined via Fisher’s Exact Test. Note that VMPs were not dichotomised into those with increased or decreased variance, as this produced an insufficient number of CpGs for statistical analysis. **(b)** As in **(a)**, except analysing DMPs associated with psychosis.


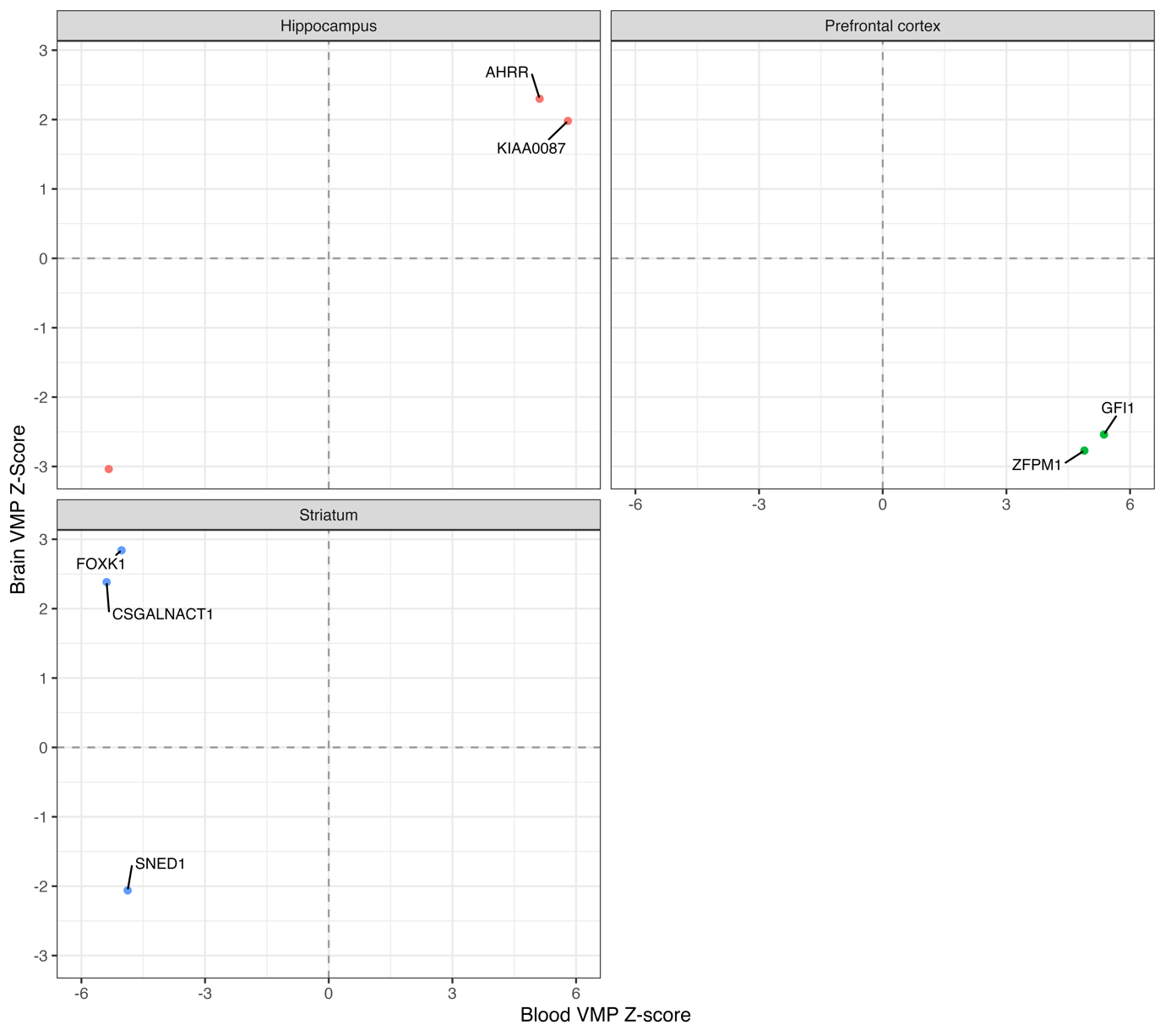


**Figure S16. Expression of psychosis associated VMPs in post-mortem brain.** Scatter plots depicting psychosis associated VMPs from blood and their corresponding variance *Z*-scores in association with schizophrenia in the hippocampus, prefrontal cortex, or striatum. All brain *Z*-scores surpassed a nominal *P* < 0.05. No psychosis associated VMPs exhibited nominal changes in the cerebellum.


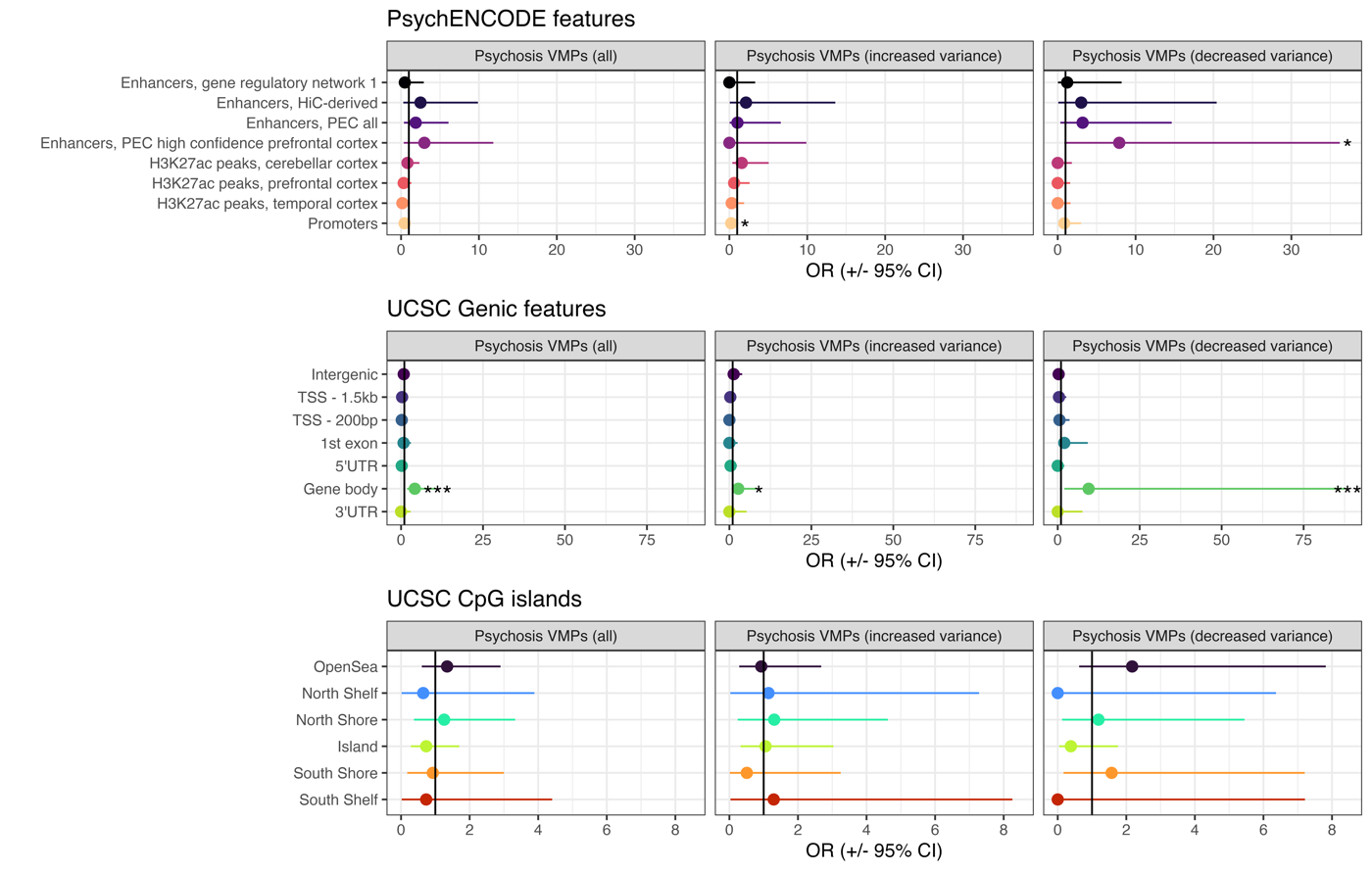


**Figure S17. Genomic enrichment profiles for psychosis associated VMPs.** Forest plots depicting enrichment of psychosis associated VMPs within genomic regulatory regions reported by *PsychENCODE* [5], gene-centric features from UCSC [6] and CpG islands and neighbouring regions from UCSC. Data presented as odds ratios ± 95% confidence interval. * = *P* < 0.05, ** = *FDR_BH_* < 0.05, *** = *P_Bonf_* < 0.05. Note that psychosis VMPs with increased or decreased variance respectively contained only 18 and 13 CpGs, respectively, thus these results should be interpreted with caution.

**
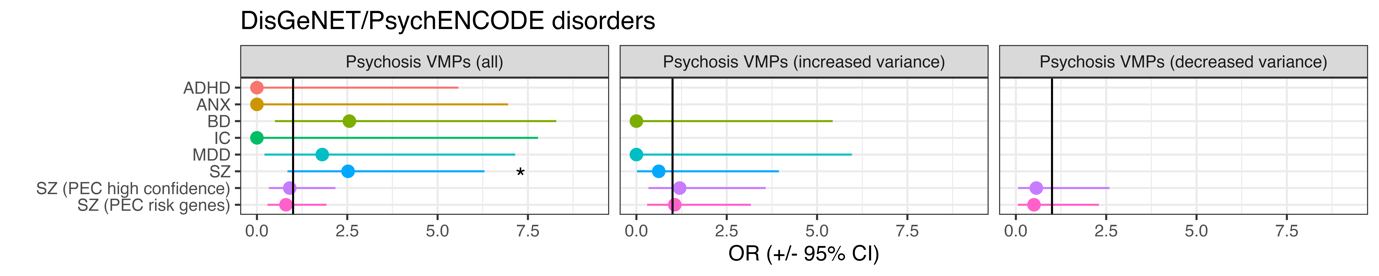

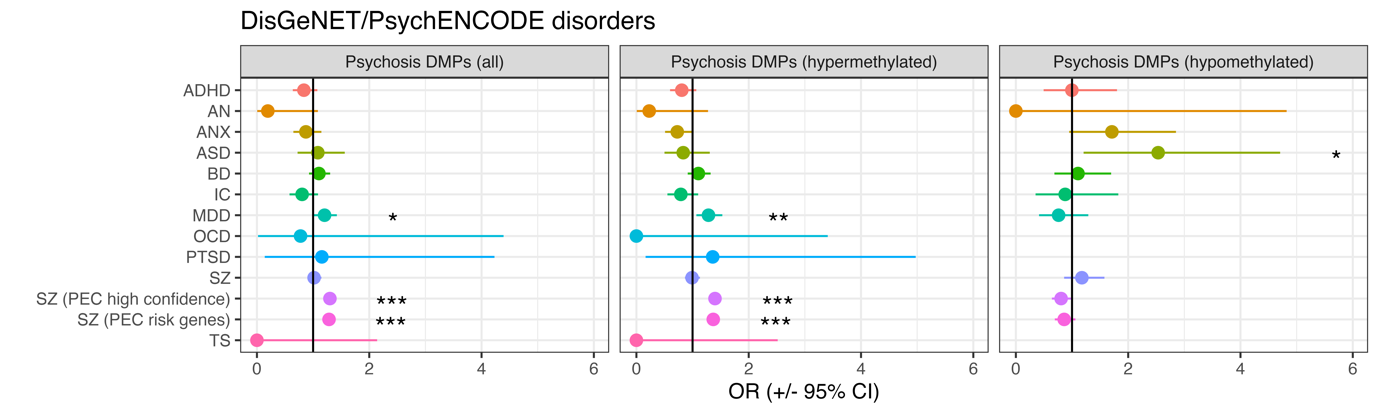
**

**(b)**

**(a)**

**Figure S18. Enrichment of psychosis associated VMPs and DMPs within genes previously associated with psychiatric illness.** Enrichment of psychosis VMPs amongst genes associated with psychiatric conditions, as defined by *DisGeNET* [15] curated and inferred gene-disorder pairings, and *PsychENCODE* [5]. Data presented as odds ratios ± 95% confidence interval. * = *P* < 0.05, ** = *FDR_BH_* < 0.05, *** = *P_Bonf_* < 0.05. ADHD = attention deficit hyperactive disorder, AN = anorexia nervosa, ANX = anxiety, ASD = autism spectrum disorders, BD = bipolar disorder, IC = impaired cognition, MDD = major depressive disorder, OCD = obsessive compulsive disorder, PTSD = post-traumatic stress disorder, SZ = schizophrenia, TS = Tourette syndrome, PEC high confidence and risk gene sets denote schizophrenia associated genes from *PsychENCODE*. Note that psychosis VMPs with increased or decreased variance respectively contained only 18 and 13 CpGs, respectively, thus these results should be interpreted with caution. **(b)** As in **(a)**, except analysing psychosis associated DMPs.

**Figure S19. Phenome-wide trait associations for methylation quantitative trait loci associated with variably methylated loci in schizophrenia.** To explore the interplay between variable DNA methylation and common genetic variants, methylation quantitative trait loci (mQTLs) significantly associated with VMPs (*P_mQTL_* < 5x10^–8^) were identified using *GoDMC* [9], after which phenome-wide association analyses (pheWAS) were conducted to examine traits related to these same genetic variants. **(a)** Amongst 66 *cis*-acting mQTLs retained for further analysis, 15 significant (*FDR_BH_* < 0.05) mQTL-trait associations were uncovered with respect to psychiatric conditions and mental health traits, including schizophrenia, depressive symptoms, major depression, bipolar disorder, ADHD and “seen doctor for nerves, anxiety, tension or depression”. A further 62 trait associations were additionally identified at a nominal *P_pheWAS_* < 0.05. Data presented as pheWAS *Z*-score (*β* / *SE*) for each mQTL. * = *P_pheWAS_* < 0.05, ** = *FDR_pheWAS_* < 0.05, *** = *P_pheWAS, Bonf_* < 0.05. **(b)** mQTL-trait associations broadly categorised into quantitative traits and other phenotypes, diseases and disorders, neuroimaging phenotypes, and biochemical traits (such as blood cells, enzymes, nutrients, vitamins and metabolites, amongst others). Data presented as mean pheWAS *Z*-score for all traits within each subcategory (full results available in Table S25).

**
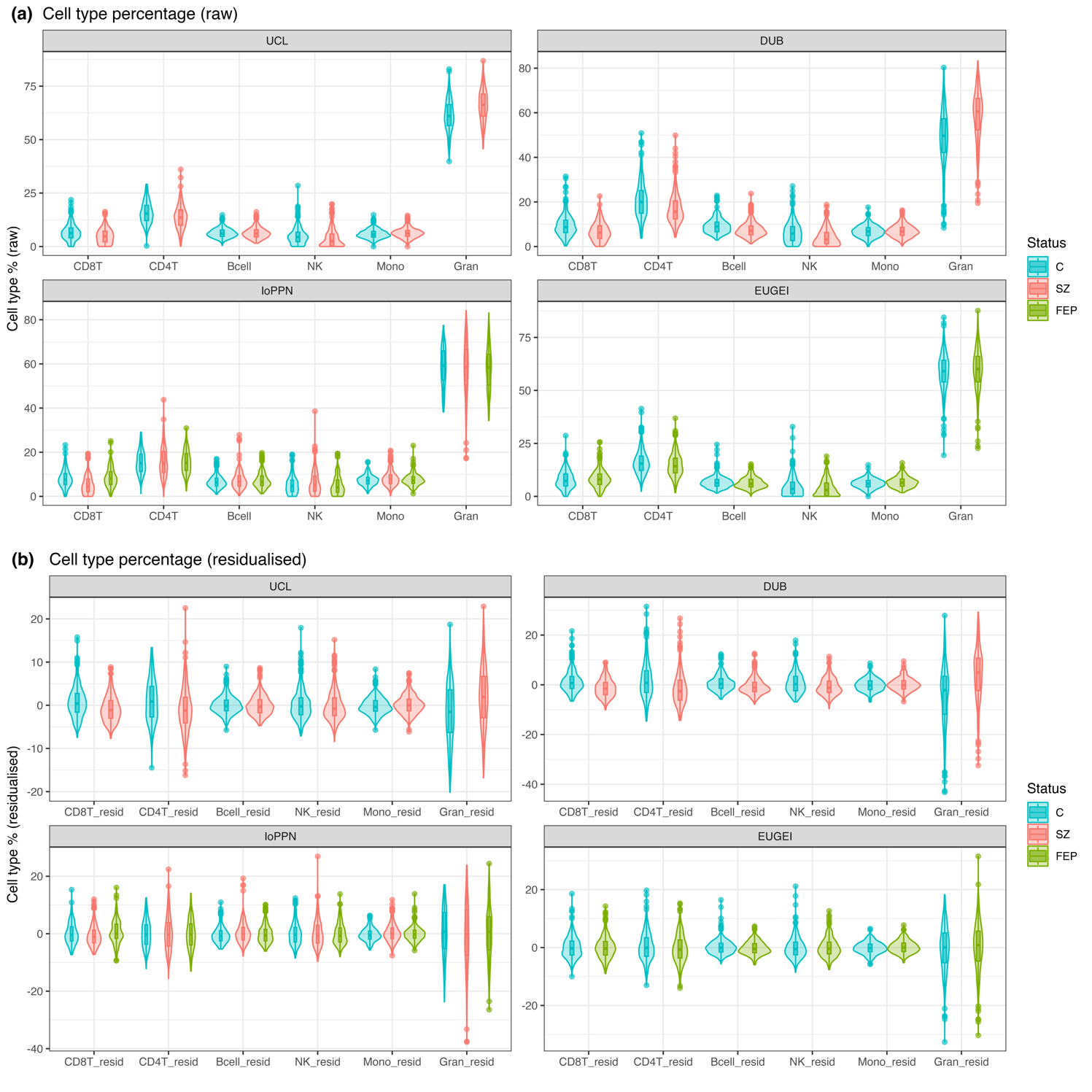
**

**Figure S20. Comparison of predicted cell-type proportions between individuals with psychiatric illness and non-psychiatric controls. (a)** Violin plots depicting raw cell-type proportions across all cohorts, predicted from a subset of CpG sites using the *wateRmelon R* library. **(b)** As in **(a)**, except after residualising cell-type proportions on predicted age, predicted sex, smoking scores, sentrix identifiers and sentrix positions. These values were utilised to examine and meta-analyse changes in the mean (multiple regression followed by IVW method) and variance (Levene’s Test followed by Stouffer’s method) of each cell-type proportion, available in Table S33. CD8T cells (*Z* = –2.16, *P* = 0.031), B cells (*Z* = 2.22, *P* = 0.027) and monocytes (*Z* = 2.57, *P* = 0.01) exhibited modest evidence for changes in variance in association with schizophrenia, while monocytes also exhibited evidence for altered variance in association with psychosis (i.e. schizophrenia and FEP diagnoses combined; *Z =* 2.41, *P* = 0.016). Monocytes cell proportions were also elevated in association with schizophrenia (*β* = 0.4, *SE* = 0.13, *P* = 1.53x10^–3^), FEP (*β* = 0.42, *SE* = 0.15, *P* = 5.02x10^–3^) and psychosis (*β* = 0.41, *SE* = 0.1, *P* = 4.98x10^–5^). Similarly, CD8T cell proportions were decreased in schizophrenia (*β* = –2.06, *SE* = 0.59, *P* = 4.4x10^–4^), while CD4T cell proportions were decreased in FEP (*β* = –0.65, *SE* = 0.33, *P* = 0.05) and psychosis (*β* = –1.99, *SE* = 0.95, *P* = 0.035).

**REFERENCES**

1. Pedersen, B.S., et al., *Comb-p: software for combining, analyzing, grouping and correcting spatially correlated P-values.* Bioinformatics, 2012. **28**(22): p. 2986-8.

2. Xu, Z., L. Niu, and J.A. Taylor, *The ENmix DNA methylation analysis pipeline for Illumina BeadChip and comparisons with seven other preprocessing pipelines.* Clin Epigenetics, 2021. **13**(1): p. 216.

3. Kolberg, L., et al., *gprofiler2 -- an R package for gene list functional enrichment analysis and namespace conversion toolset g:Profiler.* F1000Res, 2020. **9**.

4. Pei, G., et al., *deTS: tissue-specific enrichment analysis to decode tissue specificity.* Bioinformatics, 2019. **35**(19): p. 3842-3845.

5. Wang, D., et al., *Comprehensive functional genomic resource and integrative model for the human brain.* Science, 2018. **362**(6420).

6. Rosenbloom, K.R., et al., *ENCODE data in the UCSC Genome Browser: year 5 update.* Nucleic Acids Res, 2013. **41**(Database issue): p. D56-63.

7. Kiltschewskij, D.J., et al., *Alteration of DNA Methylation and Epigenetic Scores Associated With Features of Schizophrenia and Common Variant Genetic Risk.* Biol Psychiatry, 2023.

8. Seale, K., et al., *Making sense of the ageing methylome.* Nat Rev Genet, 2022. **23**(10): p. 585-605.

9. Min, J.L., et al., *Genomic and phenotypic insights from an atlas of genetic effects on DNA methylation.* Nat Genet, 2021. **53**(9): p. 1311-1321.

10. Hemani, G., et al., *The MR-Base platform supports systematic causal inference across the human phenome.* Elife, 2018. **7**.

11. Elsworth, B., et al., *The MRC IEU OpenGWAS data infrastructure.* bioRxiv, 2020: p. 2020.08.10.244293.

12. Kurki, M.I., et al., *Author Correction: FinnGen provides genetic insights from a well-phenotyped isolated population.* Nature, 2023. **615**(7952): p. E19.

13. Pidsley, R., et al., *A data-driven approach to preprocessing Illumina 450K methylation array data.* BMC Genomics, 2013. **14**: p. 293.

14. Hannon, E., et al., *DNA methylation meta-analysis reveals cellular alterations in psychosis and markers of treatment-resistant schizophrenia.* Elife, 2021. **10**.

15. Pinero, J., et al., *The DisGeNET knowledge platform for disease genomics: 2019 update.* Nucleic Acids Res, 2020. **48**(D1): p. D845-D855.
